# Modified Mediterranean Diet for Enrichment of Short Chain Fatty Acids: Potential Adjunctive Therapeutic to Target Immune and Metabolic Dysfunction in Schizophrenia?

**DOI:** 10.3389/fnins.2017.00155

**Published:** 2017-03-27

**Authors:** Jamie Joseph, Colin Depp, Pei-an B. Shih, Kristen S. Cadenhead, Geert Schmid-Schönbein

**Affiliations:** ^1^Department of Psychiatry, University of CaliforniaSan Diego, La Jolla, CA, USA; ^2^Department of Psychology, VA San Diego Healthcare SystemSan Diego, CA, USA; ^3^Department of Bioengineering, University of CaliforniaSan Diego, La Jolla, CA, USA

**Keywords:** acetate, propionate, butyrate, inflammation, gastrointestinal, psychosis, type II diabetes, cardiovascular disease

## Abstract

Growing interest in gut and digestive processes and their potential link to brain and peripheral based inflammation or biobehavioral phenotypes has led to an increasing number of basic and translational scientific reports focused on the role of gut microbiota within the context of neuropsychiatric disorders. However, the effect of dietary modification on specific gut metabolites, in association with immune, metabolic, and psychopathological functioning in schizophrenia spectrum disorders has not been well characterized. The short chain fatty acids (SCFA) acetate, butyrate, and propionate, major metabolites derived from fermentation of dietary fibers by gut microbes, interact with multiple immune and metabolic pathways. The specific pathways that SCFA are thought to target, are dysregulated in cardiovascular disease, type II diabetes, and systemic inflammation. Most notably, these disorders are consistently linked to an attenuated lifespan in schizophrenia. Although, unhealthy dietary intake patterns and increased prevalence of immune and metabolic dysfunction has been observed in people with schizophrenia; dietary interventions have not been well utilized to target immune or metabolic illness. Prior schizophrenia patient trials primarily focused on the effects of gluten free diets. Findings from these studies indicate that a diet avoiding gluten benefits a limited subset of patients, individuals with celiac disease or non-celiac gluten sensitivity. Therefore, alternative dietary and nutritional modifications such as high-fiber, Mediterranean style, diets that enrich the production of SCFA, while being associated with a minimal likelihood of adverse events, may improve immune and cardiovascular outcomes linked to premature mortality in schizophrenia. With a growing literature demonstrating that SCFA can cross the blood brain barrier and target key inflammatory and metabolic pathways, this article highlights enriching dietary intake for SCFA as a potential adjunctive therapy for people with schizophrenia.

## Schizophrenia

Schizophrenia is classically defined as a neurodevelopmental psychiatric disorder (Lewis and Levitt, 2002). However, the heterogeneous illness presentation, course, and outcomes have hindered the development of novel effective treatments. Schizophrenia is also a neuropsychiatric disorder that results in significant economic burden (Chong et al., 2016), caregiver responsibility (Szkultecka-Debek et al., 2016), and global health disability, since most patients are unable to achieve complete functional recovery (e.g., consistent paid employment, living independence, etc.).

Characteristic symptoms of schizophrenia include positive (delusions, auditory, and visual hallucinations) (Morris et al., 2012), negative (amotivation, anhedonia, apathy, inappropriate affect) (Rabinowitz et al., 2012), and dysfunction in multiple neurocognitive domains including attention (Fioravanti et al., 2005), learning and memory (Goldman-Rakic, 1994; Gold et al., 1997; Manoach, 2003), executive functioning (Hutton et al., 1998), processing speed (Rodriguez-Sanchez et al., 2007), and IQ (Zammit et al., 2004). More recent studies have consistently reported deficits in additional neuropsychiatric phenotypes including social cognition and functioning (Nuechterlein et al., 2004; Fett et al., 2011), prediction error and reward learning (Kapur, 2003; Corlett et al., 2007), and sensory gating (Braff and Geyer, 1990; Hazlett et al., 2015).

The etiology of schizophrenia resembles most chronic diseases, an interaction of complex environmental and genetic risk factors. Psychosocial stressors including poor socioeconomic status (Werner et al., 2007), migration status (Cantor-Graae and Selten, 2005), lack of social relationships (Jones et al., 1993; Schenkel et al., 2005), in conjunction with multiple genetic risk loci (Ripke et al., 2014) and epigenetic modifications (Roth et al., 2009) leads to alterations in neurotransmitter systems (Tsai et al., 1995; Howes and Kapur, 2009), neuroimmune activation (Bayer et al., 1999), brain structure (Suddath et al., 1990; Ho et al., 2003), and brain functional connectivity (Gur et al., 1985; Andreasen et al., 1994).

## Schizophrenia and attenuated lifespan

Publications implicating premature mortality as a characteristic of schizophrenia have been ongoing in the literature for the past few decades. Some of these past reports have suggested that attenuated lifespan may be independent of schizophrenia symptom chronicity. During the 1960's the primary causes of mortality in schizophrenia were thought to be due to the following diseases or illnesses: infection, cardiovascular-renal, neoplasm, endocrine and metabolic, suicide/accident or other external causes, and other disease and unspecified causes (Niswander et al., 1963).

Current estimates suggest that the lifespan for people with schizophrenia is approximately 28.5 years shorter than the general population (Olfson et al., 2015). Interestingly, the marked increase in mortality for people with schizophrenia continues to largely be a consequence of immune or metabolic illness exacerbation (Saha et al., 2007) as reported decades ago. Most recent studies suggest that cardiovascular disease (Hennekens et al., 2005; Olfson et al., 2015), type II diabetes mellitus (Olfson et al., 2015), sepsis (Seeman, 2007; Olfson et al., 2015), gastrointestinal or digestive disease (Dickerson et al., 2016), autoimmune disorders (Dickerson et al., 2016), influenza and pneumonia (Olfson et al., 2015) are the major causes of mortality in schizophrenia. To further complicate matters, these immune and metabolic disorders have complex convergent and divergent biological mechanisms (Hotamisligil, 2006). Notably, the gut is the key functional organ for many of these disorders.

## Schizophrenia and gut based dysfunction

Comparatively little scientific effort has been focused on modifying gut-neuropsychiatric pathways in schizophrenia. This is in part due to our limited knowledge of gastrointestinal (GI) functioning in relation to schizophrenia disease onset, illness course, or comorbidities. The extant literature suggests that multiple immune and metabolic makers are likely to mediate the relationships between gut functioning and neuropsychiatric outcomes.

Findings from studies of chronic schizophrenia and bipolar patients indicate serum elevation of bacterial markers (Severance et al., 2013) also present on gut microbes, implicating increased bacterial translocation from the gut. *Toxoplasma gondii* (bacteria that infects the GI tract) seropositive status has been linked to development or progression of multiple neuropsychiatric diseases, including schizophrenia (Severance et al., 2016b). Inflammatory GI diseases, especially colitis, are thought to be highly prevalent (in over 90% of samples) in schizophrenia based on post mortem biopsy (Hemmings, 1990, 2004). Sex specific GI dysfunction may also be present in males with schizophrenia due to *Candida albicans* exposure (Severance et al., 2016a).

The relationships among schizophrenia illness, diet, and gut based immune function, is also thought to be present through the c1q component of the complement pathway (Severance et al., 2012). Notably, the complement pathway makers and associated genes are recognized as potential predictors of schizophrenia genetic risk (Sekar et al., 2016), excessive synaptic pruning (Inta et al., 2016), and other biological outcomes (Nsaiba et al., 2015). Taken together, these findings support the role of diet as a possible environmental factor that contributes to the biochemical and genetic variation observed in schizophrenia. Moreover, dietary intake patterns and dietary interventions are increasingly being explored for their ability to reduce inflammation and metabolic disease risk, with a minimal likelihood of adverse effects. Therefore, these and other gut based treatments that have the potential to target converging immune and metabolic pathways could be most beneficial for people with schizophrenia.

## Article objectives

The remainder of this article will provide an overview of key observations and treatments associated with metabolic syndrome and type II diabetes, cardiovascular disease, and inflammation as pertinent to schizophrenia. This is followed by a brief analysis of dietary intake and the hypothesized neurobiological mechanisms for unhealthy dietary intake patterns in schizophrenia. Empirically based dietary modifications that have been tested in schizophrenia or are potentially relevant to schizophrenia, short chain fatty acids (SCFA), immune, and metabolic dysfunction will be reviewed. Then, the production of SCFA in the colon, their systemic transport, and findings of the SCFA in the brain and links to immune and metabolic function most germane to schizophrenia will be examined. Lastly, alternative dietary modifications, such as a high-fiber, Mediterranean style diet, that enriches production of SCFA, will be discussed as a potential adjunctive treatment for schizophrenia.

## Schizophrenia, metabolic syndrome, and type II diabetes

Metabolic syndrome is a combination of three of the following physiological factors: (1) abdominal obesity, (2) high triglyceride levels, (3) elevated high density lipoprotein (HDL) levels, (4) blood pressure, and (5) insulin resistance. It is well recognized that the incidence of metabolic syndrome, along with the incidence of type II diabetes that typically follows metabolic syndrome, is 20% higher in chronic schizophrenia patients that the general population (Dixon et al., 2000; Mitchell et al., 2013). Metformin is now being investigated and implemented as an adjunctive therapy to help mitigate antipsychotic induced metabolic syndrome (Jarskog et al., 2013).

Although, certain antipsychotic medications (McEvoy et al., 2005; De Hert et al., 2006), illness chronicity, lifestyle habits such as diet, smoking, etc., and aging related factors (Subramaniam et al., 2003) contribute to incidence of metabolic syndrome; a higher prevalence of metabolic syndrome and type II diabetes during early stages of (Correll et al., 2014) and antipsychotic medication naïve (Ryan et al., 2003; Fernandez-Egea et al., 2009; Pillinger et al., 2017) schizophrenia patients has been reported. Besides the profound effects metabolic syndrome and type II diabetes have on premature mortality in schizophrenia, they have also been associated with poor school performance in adolescence (de Nijs and Pet, 2016), sensory gating deficits (Micoulaud-Franchi et al., 2015) and other neurocognitive outcomes (Lindenmayer et al., 2012; Goughari et al., 2015).

The metabolic syndrome risks associated with schizophrenia may not be limited to factors that are observed post illness onset. Maternal type II diabetes is considered a risk factor for fetal neurodevelopment disorders, including schizophrenia (Cannon et al., 2002). The hypothesized biological mechanism for this risk factor is altered docosahexaenoic acid (DHA) transfer to the fetus (Judge et al., 2016). Therefore, it is not only important to address metabolic syndrome and diabetes in individuals who have already been diagnosed with schizophrenia, but to manage metabolic syndrome and type II diabetes in pregnant women to reduce the subsequent fetal neurodevelopmental risk. Notably, the SCFA butyrate has been shown to decrease metabolic impairments in pregnant mice (Li et al., 2013). Future studies should also consider the potential fetal neuroprotective effects of SCFA in models of maternal diabetes and risk for psychosis.

## Schizophrenia and cardiovascular disease

The etiology of cardiovascular disease as relevant to schizophrenia is multifactorial and complex, with antipsychotic medications (Peet, 2004), increased incidence of smoking (McCreadie, 2003; Hennekens et al., 2005), unhealthy dietary and excess sodium intake (Brown et al., 1999; Teasdale et al., 2016), sedentary behavior (McCreadie et al., 1998), each having a substantial role. The primary cardiovascular disease risk markers that are routinely investigated in schizophrenia include metabolic syndrome, Framingham 10-year Relative Risk score, C-reactive protein, and dyslipidemia.

The Framingham 10-year relative risk score is a recognized tool for predicting future coronary events and has been validated in various populations (Lakoski et al., 2007). Although, modifications of the Framingham 10-year relative risk score calculator have been developed for various research and treatment programs, the commonly recognized calculator is comprised of the following factors: sex, age, HDL, and total cholesterol levels, smoking status, and systolic blood pressure. Compared to individuals without a psychiatric disorder, the Framingham 10-year relative risk score is significantly higher in people with schizophrenia (Goff et al., 2005; Jin et al., 2011).

C reactive protein (CRP), an acute phase protein and cardiovascular disease risk marker (Ridker, 2001), triggers other detrimental cardiovascular outcomes such as increased clotting, generation of oxygen radicals and plaque destabilization (Prasad, 2006). CRP levels are modulated by the inflammatory cytokines Interleukin-6 (IL-6) and Tumor Necrosis Factor-α (TNF-α) that is secreted by macrophages and adipose cells (Puglisi and Fernandez, 2008). Notably, elevated CRP levels have been reported in schizophrenia by multiple research groups (Dickerson et al., 2013; Sicras-Mainar et al., 2013).

Although, cross sectional survey in the general population indicates a positive relationship between plasma CRP levels and Framingham 10-year Relative Risk score (Albert et al., 2003), the exact relationship between Framingham relative risk and CRP levels in schizophrenia remains unclear. Some research groups have found a positive association between elevated CRP levels and Framingham risk score (Sicras-Mainar et al., 2013), whereas other reports did not observe a significant association (Joseph et al., 2015). In schizophrenia, both CRP and Framingham risk have been linked to higher body mass index (BMI) (Miller et al., 2014; Joseph et al., 2015), psychiatric symptom severity (Barzilay et al., 2016; Dimitrov et al., 2016), and other dysregulated metabolic factors including fasting glucose and hemoglobin A1c levels (Dieset et al., 2012; Joseph et al., 2015). A correlation between CRP levels and routinely prescribed antipsychotic medication treatments has also been observed by some groups (Stefanovic et al., 2015). However, these findings have not been consistent as other recent findings suggest that antipsychotic medications do not target elevated CRP levels in schizophrenia (Fernandes et al., 2016).

In addition to elevated Framingham relative risk scores and CRP levels, prominent dyslipidemia has been observed in schizophrenia (Hennekens et al., 2005; Nasrallah et al., 2006). In people experiencing early illness stages of schizophrenia, significant elevations of triglycerides and other non-HDL lipids has been reported (Correll et al., 2014). In chronic patients, lipid levels have been linked to psychiatric symptom severity (Solberg et al., 2015). Pilot studies of pravastatin and simvastatin, lipid lowering medications, have been conducted in schizophrenia patients. The preliminary findings from these trials also indicate these medications also provide a temporary reduction in inflammation, positive, and negative symptoms (Chaudhry et al., 2014; Vincenzi et al., 2014). However, larger scale clinical trials that consider their long-term effects are necessary to replicate and confirm long-term beneficial outcomes.

Overall, treatment for dyslipidemia, diabetes, and hypertension has been underutilized in schizophrenia (Nasrallah et al., 2006). It is important to implement existing treatments and develop novel interventions directed at reducing cardiovascular disease risk at early illness stages to improve lifespan and outcomes in schizophrenia. Therefore, high fiber diets (Ma et al., 2006) and pharmacological interventions to target cardiovascular risk factors such as novel anticoagulants, lipid lowering agents, beta-adrenoreceptor antagonists, and angiotensin converting enzyme (ACE) inhibitors (Prasad, 2006) are important adjunctive treatments to consider for people with schizophrenia.

## Schizophrenia and inflammation

### Peripheral inflammation

Multiple immune pathways that accompany systemic inflammation are dysregulated in schizophrenia. The etiology of the dysregulated immune activity in schizophrenia has been linked to multiple causes including maternal infection, genetics, psychosocial stressors, and other environmental factors (Muller et al., 2015). The inflammatory cascade serves as a basic tissue repair mechanism (Schmid-Schonbein, 2006). Therefore, presence of inflammatory markers indicates that a tissue injury mechanism is active. However, the source of systemic inflammation in schizophrenia remains unclear as high fat diet, obesity, smoking, unhealthy diet, bacterial or viral infection, and comorbid autoimmune and gastrointestinal disorders are all likely to have a contributing role in subsets of symptoms. However, few studies consider all the above factors when investigating the role of peripheral inflammation in schizophrenia. Meta-analyses of 40 studies suggests that interleukin-1β (IL-1β), interleukin-6 (IL-6), and tumor growth factor- β (TGF-β) are cytokines associated with acute symptom exacerbation whereas TNF-α, interleukin-12 (IL-12), interferon- γ (IFN-γ), and soluble IL-2 receptor (sIL-2R) levels appear to remain stable in schizophrenia (Miller et al., 2011). Recent meta-analyses of case control studies investigating cytokine genes (Hudson and Miller, 2016), indicates polymorphisms in IL-1β, IL-6, and soluble IL-6 receptors (sIL6R) are also associated with risk for schizophrenia.

### Neuroinflammation

Elevated peripheral inflammation is closely linked to neuroinflammation in schizophrenia and other neuropsychiatric disorders (Hong et al., 2016). Inflammatory cytokines in blood can cross and interact with astrocytes comprising the blood brain barrier (Banks et al., 1995; Verkhratsky et al., 2016), circulate into the brain, and activate microglia (Norden et al., 2016). Chronic microglia activation causes a subsequent cascade of inflammatory cytokine activation in the brain that has been linked to aging brain phenotypes (Norden et al., 2015). Both protein and mRNA levels of IL-1β, TNF-α, and microglial markers were significantly increased in postmortem schizophrenia brains in relation to the brains of comparison subjects (Rao et al., 2013). Microglial activation is also elevated in people with and ultra-high-risk for schizophrenia in relation to matched comparison subjects (Bloomfield et al., 2016), with increased microglial activation primarily being observed in the hippocampus (Doorduin et al., 2009). The role of inflammatory cytokines, astrocytes, microglia, and developmental factors that can lead to neuroinflammation in psychiatric disorders has been extensively reviewed see (Meyer, 2013; Monji et al., 2013; Na et al., 2014; Verkhratsky et al., 2016).

### Treatments for inflammation

Nonsteroidal anti-inflammatory drug (NSAID) treatment trials have been conducted to target peripheral and neuroinflammation. Besides NSAIDs, minocycline (tetracycline antibiotic), raloxifene (estrogen antagonist), and N-acetylcysteine (antioxidant precursor) trials are currently underway to target elevated systemic inflammation in schizophrenia (Kianimehr et al., 2014). However, their mechanisms for targeting inflammation vary. The primary therapeutic efficacy of NSAIDs for systemic inflammation is via inhibition of cyclooxygenase-2 (COX-2) (Vane and Botting, 1998). Minocycline administration inhibits lipopolysaccharide (LPS)-induced inflammatory cytokines, major histocompatibility complex (MHC) II, and Toll-like-receptor (TLR)-2 surface expression on microglia cells (Garrido-Mesa et al., 2013). Raloxifene lowers serum levels of IL-6 and TGF-β1 and TNF-α (Ozmen et al., 2007). N-acetylcysteine modulates the nuclear factor (NF)-κB associated apoptotic pathway and inflammatory cytokine (IL-1β, IL-6, TNF-α) levels (Berk et al., 2013).

### Inflammation treatments and schizophrenia phenotypes

Many of the abovementioned treatments are thought to target schizophrenia relevant phenotypes. Specifically, oral administration of minocycline reverses altered gut microbes in hypertension and stress animal models (Wong et al., 2016) to a composition that is thought to be characteristic of a healthy gut. Minocycline, is effective at inhibiting microglial activation at adult stages, whereas it reduces synaptic pruning and neurogenesis through microglial inhibition during developmental stages (Inta et al., 2016). This suggests that minocycline may be a brain developmental stage specific treatment for neuroinflammation observed schizophrenia. Raloxifene has demonstrated additional benefits in schizophrenia including general psychopathology reduction (Kulkarni et al., 2016; Usall et al., 2016), improvements in attention and memory (Weickert et al., 2015), and increased activation in the right hippocampus and left inferior frontal gyrus (Ji et al., 2016). N-acetyl cysteine has also demonstrated benefits in psychopathology symptom reduction (Berk et al., 2013).

Outcomes from prior NSAID trials in schizophrenia patients suggest low efficacy for celecoxib and inconsistent findings for aspirin (Sommer et al., 2013). As noted earlier, the estimated prevalence of inflammatory GI disorders in schizophrenia is high (Hemmings, 2004), thereby rendering NSAIDs an unsuitable treatment (Sigthorsson et al., 1998). The number of and findings from minocycline, raloxifene, and N-acetyl cysteine trials remains too few and preliminary to determine whether these treatments will be effective long-term adjunctive treatment strategies. In addition, these medications do not target the underlying cause or primary source of peripheral inflammation, i.e., tissue injury, in schizophrenia, which remains unresolved. Modified Mediterranean dietary adaptations for enrichment of SCFA may have great therapeutic value to target inflammation in schizophrenia. SCFA and specific dietary modifications (Urpi-Sarda et al., 2012) have demonstrated ability to lower gut and systemic inflammation.

## Schizophrenia, obesity, and unhealthy dietary intake

Numerous clinical and epidemiological studies indicate that the rates of obesity and morbid obesity for people with schizophrenia are significantly higher than the general population (Hsiao et al., 2004; Susce et al., 2005). While elevated BMI is commonly associated with second generation antipsychotic medication effects (Grunder et al., 2016); ethnic/racial differences, diet, physical activity, and other lifestyle factors also contribute to the prevalence of obesity in schizophrenia (Brown et al., 1999; Norlelawati et al., 2012).

Current studies of dietary intake in schizophrenia suggest that most patients have a poor diet largely characterized by increased fast and processed foods (Strassnig et al., 2003), increased sodium and cholesterol intake (Nunes et al., 2014), and higher saturated fat and lower fiber content than non-psychiatric comparison subjects (Brown et al., 1999; Henderson et al., 2006). Although, increased sugar and processed diet consumption is thought to be characteristic of people with schizophrenia originating from Western and European countries (Peet, 2003; Stokes and Peet, 2004), similar findings have also been observed in patients from Eastern countries (Sugawara et al., 2014; Ito et al., 2015). The interaction of sex specific effects for BMI and dietary consumption in schizophrenia may also be present with female patients reported to have a higher BMI and unhealthier diet than males (Amani, 2007; Carliner et al., 2014). Homelessness and poor socioeconomic status is also likely to have a major influence on nutritional status and dietary intake patterns in schizophrenia. However, this has not been taken into consideration for many studies.

Nutritional changes including ω-3 or vitamin D deficiencies (Dealberto, 2007) may increase risk for developing psychosis. A lower intake of ω-6, phytosterols, vitamin A, and vitamin E (α-tocopherol) has also been reported in relation to BMI and demographically matched comparison subjects (Nunes et al., 2014). In addition, consumption of fruit and vegetable portions is significantly lower that the recommended daily allowance (Heald et al., 2015). However, other reports indicate that it is primarily the overall, rather than specific type of, caloric intake that is increased in schizophrenia (Strassnig et al., 2003). Additional scientific investigations are needed to clarify the relationships between lifestyle factors, dietary intake, and nutritional status in schizophrenia patient populations. Elucidating the specific role of dietary compounds and nutritional factors in relation to symptom and behavioral outcomes will require combined efforts from animal model testing and human clinical trials.

## Schizophrenia and neurobiology of unhealthy dietary intake

The neurobiological and neurocognitive mechanisms that lead to unhealthy dietary intake by people with schizophrenia are hypothesized to be related to dysregulated reward circuitry (Elman et al., 2006): a hyperdopamineric mesolimbic pathway combined with poor cognitive control (Kapur, 2003). This pathway has also been implicated in the context of obesity (Vucetic and Reyes, 2010), food cravings (Blum et al., 2011), eating disorders (Wagner et al., 2007) and addiction (Volkow et al., 2012), with altered reward circuitry being a major common pathway linked to comorbid substance abuse in schizophrenia (Chambers et al., 2001).

Deficits in reward learning have been observed in multiple schizophrenia patient studies (Juckel et al., 2006) and have been consistently linked to co-occurring negative symptoms (Strauss et al., 2011; Gold et al., 2012). Yet, the ability to alter responses with the use of prediction error is thought to be intact for people with schizophrenia (Gold et al., 2012). This could potentially be utilized as a cognitive strategy to help modify and improve the quality of dietary intake. However, the specific relationships between reward circuits and dietary intake patterns in schizophrenia remain unclear. Novel investigations are needed to determine the neurocognitive and neurobiological factors that may be contributing to unhealthy dietary intake and nutritional status in schizophrenia.

## Schizophrenia, empirically based diets, and nutritional supplements

### Gluten free diets

Celiac disease is an autoimmune disorder that results in inflammatory intestinal damage after the ingestion of food products containing gluten including wheat, barley, bulgur, rye, and seitan. Gluten exposure, for individuals with celiac disease, results in the increase in the expression of HLA antigen markers on cells in the surface layers of the intestinal mucosa. T cells react to this and subsequently release IFN-γ, TNF-α, and other cytokines (Murray, 1999). The incidence of celiac disease and non-celiac gluten sensitivity (Singh and Kay, 1976; Eaton et al., 2006; Cascella et al., 2009; Okusaga et al., 2013) is higher in schizophrenia that the general population. Celiac disease and non-celiac gluten sensitivity coincide with elevated levels of anti-tissue transglutaminase and anti-gliadin antibodies, respectively (Cascella et al., 2009, 2013). Increased IgG responses to gluten have also been associated with the activation of additional immune pathways dysregulated in schizophrenia such as complement C1q (Severance et al., 2012) in schizophrenia.

Selected case reports and follow up studies indicate that psychotic symptoms can be triggered by gluten in those with a gluten intolerance (Lionetti et al., 2015). However, comorbid non-celiac gluten sensitivity or celiac disease has not been consistently linked to exacerbation of psychopathology (Cascella et al., 2009; Jackson et al., 2014). Although, a few studies have demonstrated that gluten free diets lead to improved symptoms (Dohan and Grasberger, 1973; De Santis et al., 1997; Jackson et al., 2012), replication studies have yielded mixed results. In addition, consumption of a gluten free diet in individuals who did not have a diagnosis of celiac disease or non-celiac gluten sensitivity led to lower butyrate levels, reductions in beneficial gut microbial species, and increased host immune activation (De Palma et al., 2009). The observed findings may also be accounted for by decreased intake of fermentable fiber leading to reduced production of SCFA. Therefore, the implementation of gluten free diets for schizophrenia patient treatment may be best limited to those individuals it is most likely benefit; people with non-celiac gluten sensitivity or celiac disease (Kalaydjian et al., 2006).

### Omega 3 fatty acid supplementation

Omega-3 (ω-3) fatty acids are polyunsaturated fatty acids involved in cellular metabolism (von Schacky et al., 1985). ω-3 fatty acids, or their precursor α-linolenic acid, cannot be synthesized by humans and must be derived from dietary sources (Harris et al., 2008). The major dietary sources of ω-3 fatty acids are seafood, poultry, and eggs. α-linolenic acids are largely found in nuts, soybean, canola, and flax seed oils (Innis, 2008). The ω-3 fatty acids with prominent immune and metabolic functions include docosahexaenoic acid (DHA), docosapentaenoic acid (DPA), and eicosapentaenoic acid (EPA).

ω-3 fatty acids bind to the free fatty acid receptors FFA1 (GPR40) and FFA4 (GPR120). GPR40 receptors are expressed in pancreatic beta cells and regulate insulin secretion (Itoh et al., 2003). DHA acts on GPR120 receptors in macrophages and adipocytes to mediate anti-inflammatory and insulin sensitizing effects (Oh et al., 2010). Fatty acids also reduce the growth of the atherosclerotic plaque via reduction in interleukin 1 (IL-1) and TNF-α and by inhibiting the migration of monocytes (Zamaria, 2004). Trials of ω-3 fatty acids as a means to improve cardiovascular health have been effective in reducing sudden cardiac death (Mozaffarian and Wu, 2011). However, the effects of ω-3 treatment on other cardiovascular outcomes are not as clearly delineated. These and other potential relationships between ω-3 intake and cardiovascular disease risk such as dyslipidemia should be further examined.

Overall, clinical trials of ω-3 fatty acids in schizophrenia have demonstrated improvement in psychopathology (Peet, 2006). Studies comparing EPA and DHA in schizophrenia primarily indicate symptom improvement with EPA and not DHA (Peet et al., 2001). This is consistent with the findings for the roles of EPA and DHA in most mood disorders (Dyall, 2015) implicating a specific role for EPA in neuropsychiatric function. Additional reports suggest that ω-3 fatty acids are helpful in reducing tardive dyskinesia in schizophrenia patients (Emsley et al., 2002). A recent meta-analyses of randomized trials of ω-3 supplementation in schizophrenia reported attenuated risk of conversion to psychosis in prodromal patients (Chen et al., 2015). In first-episode studies, ω-3 fatty acids decreased non-psychotic symptoms and improved treatment response rates (Chen et al., 2015). However, findings from stable chronic schizophrenia patients are mixed (Chen et al., 2015).

DHA is the primary ω-3 fatty acid in the brain (Dyall, 2015). Although, this is counterintuitive to the findings of beneficial effects for EPA rather than DHA or DPA in schizophrenia and mood disorder clinical trials; significantly lower DHA concentrations have been observed in the orbitofrontal cortex of postmortem schizophrenia brains relative to age and gender matched comparison brains (McNamara et al., 2007). The higher concentration of DHA observed in the healthy brain may be linked to its critical roles in enhancing neurogenesis, neurite outgrowth, and synaptogenesis (Cao et al., 2009). Administration of EPA + DHA reversed age related reduction in glutamate receptors GluR2 and NR2B in rodents (Dyall et al., 2007). This suggest that ω-3 fatty acids may have a key role in synaptic plasticity and hippocampal glutamatergic transmission that may be of relevance to the schizophrenia postmortem findings (McNamara et al., 2007) and other relevant neurobiological observations (Gao et al., 2000). EPA has also demonstrated modulatory effects on neurotrophin receptors (Kou et al., 2008). This may potentially be linked to the observations of reduced prefrontal cortical expression of neurotrophins in schizophrenia (Weickert et al., 2005). Follow up studies that account for potential neurotransmitter and neurotrophic interactions are needed to clarify the predictors of positive or negative response EPA, DHA, or DPA in schizophrenia patients across illness course and lifespan.

### Ketogenic diets

Ketogenic diets are a high-fat, low-carbohydrate, diet routinely administered to manage treatment refractory epileptic seizures (Kinsman et al., 1992). Trials of ketogenic diets in non-psychiatric populations have established their efficacy for short term weight loss (Foster et al., 2003). Investigation of ketogenic diets in schizophrenia patient populations have been limited to a case (Kraft and Westman, 2009) and small pilot study (Pacheco et al., 1965) with female schizophrenia patients. Consumption of a ketogenic diet for 2 weeks resulted in improvement in behavioral symptoms that returned 1 week after discontinuing the diet (Pacheco et al., 1965).

In a mouse model highly susceptible to seizures (DBA/2J), ketogenic diet consumption was able to successfully target sensory gating deficits (Tregellas et al., 2015), a consistently reported neurocognitive phenotype for schizophrenia spectrum populations (Earls et al., 2016). In addition, Kraeuter et al. (2015) demonstrated that the administration of a ketogenic diet to NMDA receptor hypofunction mouse model of schizophrenia (MK-801) normalized model induced behaviors, led to weight loss, decreased glucose levels, and elevated β-hydroxybutyrate. β-hydroxybutyrate is a metabolite utilized as an energy source during ketogenesis, has histone deacetylase inhibitor activities, and is modulated by SCFA (Selkrig et al., 2014). Notably, increased serum and urine β-hydroxybutyrate has been observed in early and chronic schizophrenia patients, implicating a role for dysregulated energy metabolism (Yang et al., 2013). Yet, β-hydroxybutyrate is also thought to have potential protective effects against brain injury and neurodegenerative diseases via activation of macrophage subsets (Rahman et al., 2014). Replication of Yang et al.'s findings and additional experiments will help determine if the elevated β-hydroxybutyrate levels in schizophrenia are a consequence of impaired energy metabolism or a compensatory neurodefense mechanism.

However, ketogenic diets can also lead to significant adverse effects that are salient for individuals with schizophrenia. The most notable of these include reduced performance on higher level cognitive tasks based on testing in overweight women (Wing et al., 1995) and long-term dysregulation of blood lipid levels that was observed in epilepsy patients (Kwiterovich et al., 2003). Therefore, the utility of ketogenic diets as an adjunctive intervention for people with schizophrenia may be limited. Moreover, since this style of diet drastically reduces or eliminates carbohydrate consumption, most ketogenic diets are also likely to be virtually gluten-free (Kraft and Westman, 2009). Larger scale, randomized, trials of ketogenic diets that account for gluten sensitivity, celiac disease, and assess metabolic, neurobiological, and behavioral endpoints, will be necessary to confirm the therapeutic value of ketogenic diets for people with schizophrenia.

### Dietary approaches to stop hypertension (DASH)

The DASH is a low-sodium, low-fat, diet that was designed with the aim to improve hypertension (Sacks et al., 2001; Blumenthal et al., 2010) and reduce the risk for ischemic stroke (Larsson et al., 2016). Similar to ketogenic diets, DASH diets have been established as an effective weight loss intervention (Miller et al., 2002). Small scale investigations of the DASH diet in obese, post-menopausal, women indicate that the DASH diet may reduce circulating propionate while increasing acetate and butyrate (Mathew et al., 2015). Meta-analyses of randomized trials suggest that DASH diets lead to greater weight loss than other types of low calorie diets (Soltani et al., 2016).

Studies of the DASH diet in relation to cardiovascular or psychopathological outcomes in schizophrenia are very limited. A similar nutritional intervention conducted in a first episode schizophrenia patient sample was effective at reducing excessive sodium and caloric intake observed at study baseline (Teasdale et al., 2016). While the DASH diet and other sodium intake reduction measures are quite effective at reducing hypertension and inflammation, compliance has been suboptimal in various clinical populations (Feyh et al., 2016; Teasdale et al., 2016). Successful implementation of the DASH or modified DASH diet to target hypertension or other cardiovascular risk factors will require cognitive and behavioral modification strategies to maintain adherence in schizophrenia spectrum populations.

## Colonic generation of short chain fatty acids and transport to the brain

The short chain fatty acids (SCFA) acetate, propionate, and butyrate are the primary metabolic products derived from the colonic fermentation of dietary fibers by gut microbes and is estimated to be produced in a molar ratio of 60:20:20 respectively (Wong et al., 2006). Table 1 summarizes the major dietary carbohydrates, primary sources for these carbohydrates, and identified gut microbiota that ferment these carbohydrates to produce SCFA.

**Table 1:** **Major fermentable dietary carbohydrates and identified gut microbes associated with colonic acetate, propionate, and butyrate production**.

**Major carbohydrate starting product**	**Primary dietary sources for carbohydrate starting product**	**Genus or species of identified fermenting microbes**
***Monosaccharides***		
Fructose	Agave nectar	*Lactobacillus* spp. *Bifidobacterium* spp. *Faecalibacterium* spp.
	Honey	
	Fruits	
Glucose^a^	Squash	Faecalibacterium spp.
	Apples	
	Raspberries	
	Peas	
***Disaccharides***		
Lactose	Milk	*Lactobacillus* spp. *Bifidobacterium* spp. *Streptococcus* spp. *Escherichia* coli
	Yogurt	
	Buttermilk	
	Cheese	
Sucrose	Sugar cane	*Lactobacillus* spp.
	Dates	
	Sugar beets	
	Sweet peas	
	Fruits	
***Oligosaccharides***		
Fructooligosaccharides^b^	Onion	*Bifidobacterium* spp.
	Chicory	
	Garlic	
	Asparagus	
	Banana	
	Artichoke	
Galactooligosaccharides^b^	Artichoke	*Bifidobacterium* spp.
	Beans	
	Beetroot	
	Broccoli	
	Chickpeas	
	Fennel	
	Lentils	
	Lettuce	
	Radicchio	
	Onion	
	Peas	
Raffinose	Cottonseed flour	*Lactobacillus* spp. *Bifidobacterium* spp.
	Soy flour	
	Onions	
	Chickpeas	
	Beans	
	Peas	
	Lentils	
Stachyose	Cottonseed flour	*Bifidobacterium* spp. *Lactobacillus* spp.
	Soy flour	
	Onions	
	Chickpeas	
	Beans	
	Peas	
	Lentils	
***Polysaccharides***		
Amylose	Potato	*Bifidobacterium* spp. *Eubacterium* spp. *Ruminococcus* spp. *Prevotella* spp.
	Corn	
	Wheat	
	Tapioca	
	Rice	
Amylopectin	Potato	*Faecalibacterium* spp. *Bifidobacterium* spp. *Collinsella* spp. *Eubacterium* spp. *Prevotella* spp.
	Corn	
	Wheat	
	Tapioca	
	Rice	
B glucan^b^	Oat	*Eubacterium* spp. *Atopobium* spp. *Enterococcus* spp. *Lactobacillus* spp. *Prevotella* spp. *Clostridium cluster* XIVa
	Barley	
	Wheat	
	Rye	
	Mushrooms	
	Seaweed	
Gum Arabic^b^	Acacia tree	*Bifidobacterium* spp.
	Prepared food additive	*Lactobacillus* spp.
		*Ruminococcus* spp.
Guar Gum	Guar bean	*Bifidobacterium* spp. *Ruminococcus* spp.
	Prepared food additive	
Inulin^b^	Asparagus	*Bifidobacterium* spp. *Faecalibacterium* spp.
	Leek	
	Onions	
	Banana	
	Wheat	
	Garlic	
Laminarin	Seaweed	*Prevotella* spp.
Resistant starch^b^	Cashew	*Roseburia* spp. *Eubacterium* spp. *Ruminococcaceae* spp.
	Green Banana	
	White Beans	
	Oat	
	Potato	
***Arabinoxylans***		
Cellulose	Seaweed	*Bifidobacterium* spp.
	Wheat bran	
Pectin	Apples	*Eubacterium* spp.
	Apricots	
	Cherries	
	Oranges	
	Carrots	
***Sugar Alcohols***		
Mannitol	Carrots	*Bifidobacterium* spp. *Lactobacillus* spp. *Streptococcus* spp. *Escherichia* spp.
	Asparagus	
	Olives	
	Sweet potatoes Pineapple	
	Mushrooms	
	Seaweed	
Sorbitol	Pear	*Lactobacillus* spp. *Streptococcus* spp. *Escherichia* spp. *Salmonella* spp. *Shigella* spp.
	Prune	
	Dried rose hip	
	Peaches	
	Cherries	
	Dried fruit mix	
	Plums	
	Dates	
Xylitol	Fruit	*Bifidobacterium* spp. *Streptococcus* spp. *Prevotella* spp.
	Mushrooms	
	Vegetables	
	Oats	
	Corn	
***Other***		
Acetate to Butyrate Conversion	Produced by microbial fermentation and contained in food products made by bacterial fermentation	*Faecalibacterium* spp. *Eubacterium* spp. *Anaerostipes* spp.
Lactate to Butyrate Conversion	Produced by microbial fermentation and contained in food products made by bacterial fermentation	*Eubacterium* spp. *Anaerostipes* spp. *Clostridium cluster* XIVa

a *Is also directly absorbed into the circulation without fermentation by gut microbiota*.

b *Is recognized to have prebiotic properties (Roberfroid et al., 2010)*.

Short chain fatty acids bind to the free fatty acid receptors FFAR2 (GPR41) and FFAR3 (GPR43). Propionate binds to human GPR41 and GPR43 with equal affinity. Acetate has shown to be selective for GPR43, whereas butyrate preferentially binds GPR41 (Le Poul et al., 2003). GPR41 expression has been detected in the autonomic sensory ganglia, pancreas, spleen, lymph nodes, bone marrow, adipose tissue, and peripheral blood mononuclear cells including monocytes (Brown et al., 2003; Le Poul et al., 2003; Nohr et al., 2015). GPR43 expression has primarily been observed in the colon, ileum, adipose tissue, and immune cells especially monocytes and neutrophils (Brown et al., 2003; Le Poul et al., 2003; Nilsson et al., 2003). Expression of these receptors in human or animal brain models has not yet been well characterized.

Most of the butyrate that is produced in the colon is taken up by colonocytes as an energy source (Wong et al., 2006). However, SCFA are also then transported from the colon via the hepatic portal vein to the liver. The predominant SCFA that is absorbed by the liver is propionate for gluconeogenesis (Cummings et al., 1987). From the liver, SCFA enter the systemic circulation. In healthy, BMI and lipid level matched, men and women, serum concentrations of SCFA ranged from 20–190, 1.7–8.4, and 0.0–7.6 μmol/L for acetate, propionate, and butyrate, respectively (Wolever and Bolognesi, 1996). Circulating levels of SCFA are able to cross the blood brain barrier, and the primary SCFA uptake by the brain is for butyrate, followed by propionate and acetate (Oldendorf, 1973). In the human brain, butyrate and propionate concentrations are estimated to be 17.0 and 18.8 pmol/mg, respectively (Bachmann et al., 1979).

## Short chain fatty acids and inflammation

Sodium butyrate is anti-inflammatory against LPS induced inflammation in rat primary microglia, hippocampal cultures, and neuronal co-cultures of microglial cells, astrocytes and cerebellar granule neurons (Huuskonen et al., 2004). Butyrate treatment in hippocampal slice cultures also resulted in the downregulation of NF-κB-binding capacity induced by LPS (Huuskonen et al., 2004).

In addition to targeting brain derived inflammation, SCFA have the ability to modulate multiple immune and epigenetic pathways including obesity induced inflammation (Meijer et al., 2010), IL-6 and TNF-α release from macrophages (Kim et al., 2014), inhibiting cytokine induced NF-κB activation (Tedelind et al., 2007), and histone deacetylase inhibition (Wong et al., 2006). These biological pathways have also been shown to be dysregulated in schizophrenia (Fan et al., 2007; Sharma et al., 2008; Song et al., 2009; Miller et al., 2011). Therefore, the potential of SCFA as an adjunctive treatment may have significant beneficial outcomes. However, the role of SCFA in schizophrenia risk, onset, and comorbid illness outcomes remains unknown.

Although, the abovementioned studies demonstrate that SCFA are present in the brain and modify inflammation in a beneficial manner, administration of valproic acid, a medication commonly prescribed for symptoms associated with bipolar disorder and epilepsy, inhibits the transport of SCFA across the blood brain barrier in rodents (Adkison and Shen, 1996). In addition, *in vitro* studies reveal that free fatty acids in the intestine can have cytotoxic properties (Penn and Schmid-Schonbein, 2008). Therefore, there is still much to be learned about the compounds that modulate SCFA and the types and expression of the receptors that SCFA target. In addition, the role of SCFA in the brain and their relationship to neurobiological factors and pathways including neurotransmitter circuits, neurotrophic factors and other brain metabolites remains largely unknown.

## Mediterranean style diets can target immune and metabolic outcomes associated with schizophrenia

Mediterranean based diet treatment trials have led to reductions in overall cardiovascular disease risk (Estruch et al., 2013) compared to most Western diets. It is thought that the Mediterranean diet primarily ameliorates cardiovascular disease by providing a more optimal ω-6/ω-3 ratio (Simopoulos, 2002). In addition, adherence to a Mediterranean style diet reduces levels of CRP and TNF-α (Koloverou et al., 2016; Neale et al., 2016), immune markers routinely linked to poor cardiovascular outcomes. In type II diabetes patients, the Mediterranean style diet significantly reduced hemoglobin A1c levels (Elhayany et al., 2010), suggesting that a Mediterranean style diet will likely benefit schizophrenia patients with comorbid type II diabetes.

In individuals who were defined as healthy based on screening for autoimmune, cancer, and digestive diseases, a higher Mediterranean diet score was associated with increased abundance of health beneficial gut microbiota and coincided with higher fecal concentrations of propionate and butyrate (Gutierrez-Diaz et al., 2016). Studies comparing the effects of Mediterranean style, ketogenic, and low fat diets in obese individuals indicate that Mediterranean diets are equally effective as the other diets for weight loss while having the added benefit of maintaining glycemic and lipid control (Shai et al., 2008). To our knowledge, studies of Mediterranean diets in schizophrenia have not yet been conducted. Therefore, incorporating a high-fiber, ω-3 rich, Mediterranean style diet into patient lifestyle management and treatment could very likely improve the metabolic and immune outcomes that have consistently linked to premature mortality in schizophrenia. Higher fermentable fiber intake through a modified Mediterranean diet lifestyle should increase circulating levels of SCFA and may also directly mitigate significant constipation, a gastrointestinal side effect associated with some commonly prescribed antipsychotic medications (De Hert et al., 2011).

## Conclusions

Dietary modifications and interventions provide an opportunity to directly target gut, immune, and metabolic markers. This remains highly underexplored in the context of psychiatric disorders, especially schizophrenia. Dietary and nutritional investigations that have been conducted in schizophrenia are few, with randomized controlled trials of dietary modification being scarce, thereby leaving a significant gap in the schizophrenia literature.

Schizophrenia patients are most likely to benefit from the implementation of individualized dietary interventions due to co-occurring nutritional deficiencies (Hoffer, 2008; Kale et al., 2010). Many people with schizophrenia vary in insight with regards to their illness (Aleman et al., 2006), dietary intake habits (Heald et al., 2015), social support (Buchanan, 1995), and everyday functioning (Sabbag et al., 2012). For dietary interventions in schizophrenia patient populations to be successful, the combined support from scientists, dieticians, family members, and neuropsychiatric clinicians who have been successful in implementing behavioral modifications will be necessary.

SCFA can be assayed from blood and fecal samples, and enriched through high fiber dietary modification. To date, the role of SCFA in relation to dietary intake, immune, and metabolic outcomes has not been investigated in schizophrenia. Therefore, it is unknown whether high-fiber, Mediterranean type, dietary modification enriched for SCFA production, could have a direct effect on improving schizophrenia psychopathology, an additive effect via concurrent modification of immune and metabolic markers that may trigger schizophrenia symptoms, or primarily an indirect effect. Translational animal studies and human clinical trials will be needed to determine the exact SCFA and Mediterranean dietary components that can improve short and long term physiological and behavioral outcomes for people with schizophrenia.

## Author contributions

JJ developed the hypotheses and wrote the manuscript. CD assisted with hypothesis development. PS, KC, and GS edited the manuscript.

### Conflict of interest statement

The authors declare that the research was conducted in the absence of any commercial or financial relationships that could be construed as a potential conflict of interest.

## References

[B1] Adkison K. Shen D. D. (1996). Uptake of valproic acid into rat brain is mediated by a medium-chain fatty acid transporter. J. Pharmacol. Exp. Ther. 276, 1189–1200. 8786552

[B2] Albert M. A. Glynn R. J. Ridker P. M. (2003). Plasma concentration of C-reactive protein and the calculated Framingham Coronary Heart Disease Risk Score. Circulation 108, 161–165. 10.1161/01.CIR.0000080289.72166.CF 12835213

[B3] Aleman A. Agrawal N. Morgan K. D. David A. S. (2006). Insight in psychosis and neuropsychological function. Br. J. Psychiatry 189, 204–212. 10.1192/bjp.189.3.204 16946354

[B4] Amani R. (2007). Is dietary pattern of schizophrenia patients different from healthy subjects? BMC Psychiatry 7:15. 10.1186/1471-244X-7-15 17474979 PMC1868716

[B5] Andreasen N. C. Arndt S. Swayze V. Cizadlo T. Flaum M. O'Leary D.. (1994). Thalamic abnormalities in schizophrenia visualized through magnetic resonance image averaging. Science 266, 294–298. 10.1126/science.7939669 7939669

[B6] Bachmann C. Colombo J. P. Beruter J. (1979). Short chain fatty acids in plasma and brain: quantitative determination by gas chromatography. Clin. Chim. Acta 92, 153–159. 10.1016/0009-8981(79)90109-8 487569

[B7] Banks W. A. Kastin A. J. Broadwell R. D. (1995). Passage of cytokines across the blood-brain barrier. Neuroimmunomodulation 2, 241–248. 10.1159/000097202 8963753

[B8] Barzilay R. Lobel T. Krivoy A. Shlosberg D. Weizman A. Katz N. (2016). Elevated C-reactive protein levels in schizophrenia inpatients is associated with aggressive behavior. Euro. Psychiatry 31, 8–12. 10.1016/j.eurpsy.2015.09.461 26657596

[B9] Bayer T. A. Buslei R. Havas L. Falkai P. (1999). Evidence for activation of microglia in patients with psychiatric illnesses. Neurosci. Lett. 271, 126–128. 10.1016/S.0304-3940(99)00545-5 10477118

[B10] Berk M. Malhi G. S. Gray L. J. Dean O. M. (2013). The promise of N-acetylcysteine in neuropsychiatry. Trends Pharmacol. Sci. 34, 167–177. 10.1016/j.tips.2013.01.001 23369637

[B11] Bloomfield P. S. Selvaraj S. Veronese M. Rizzo G. Bertoldo A. Owen D. R. (2016). Microglial Activity in People at Ultra High Risk of Psychosis and in Schizophrenia: an [(11)C]PBR28 PET Brain Imaging Study. Am. J. Psychiatry 173, 44–52. 10.1176/appi.ajp.2015.14101358 26472628 PMC4821370

[B12] Blum K. Liu Y. Shriner R. Gold M. S. (2011). Reward circuitry dopaminergic activation regulates food and drug craving behavior. Curr. Pharm. Des. 17, 1158–1167. 10.2174/138161211795656819 21492092

[B13] Blumenthal J. A. Babyak M. A. Hinderliter A. Watkins L. L. Craighead L. Lin P. H.. (2010). Effects of the DASH diet alone and in combination with exercise and weight loss on blood pressure and cardiovascular biomarkers in men and women with high blood pressure: the ENCORE study. Arch. Intern. Med. 170, 126–135. 10.1001/archinternmed.2009.470 20101007 PMC3633078

[B14] Braff D. L. Geyer M. A. (1990). Sensorimotor gating and schizophrenia: human and animal model studies. Arch. Gen. Psychiatry 47, 181–188. 10.1001/archpsyc.1990.01810140081011 2405807

[B15] Brown A. J. Goldsworthy S. M. Barnes A. A. Eilert M. M. Tcheang L. Daniels D.. (2003). The Orphan G protein-coupled receptors GPR41 and GPR43 are activated by propionate and other short chain carboxylic acids. J. Biol. Chem. 278, 11312–11319. 10.1074/jbc.M211609200 12496283

[B16] Brown S. Birtwistle J. Roe L. Thompson C. (1999). The unhealthy lifestyle of people with schizophrenia. Psychol. Med. 29, 697–701. 10.1017/S0033291798008186 10405091

[B17] Buchanan J. (1995). Social support and schizophrenia: a review of the literature. Arch. Psychiatr. Nurs. 9, 68–76. 10.1016/S0883-9417(95)80003-4 7755410

[B18] Cannon M. Jones P. B. Murray R. M. (2002). Obstetric complications and schizophrenia: historical and meta-analytic review. Am. J. Psychiatry 159, 1080–1092. 10.1176/appi.ajp.159.7.1080 12091183

[B19] Cantor-Graae E. Selten J.-P. (2005). Schizophrenia and migration: a meta-analysis and review. Am. J. Psychiatry 162, 12–24. 10.1176/appi.ajp.162.1.12 15625195

[B20] Cao D. Kevala K. Kim J. Moon H. S. Jun S. B. Lovinger D.. (2009). Docosahexaenoic acid promotes hippocampal neuronal development and synaptic function. J. Neurochem. 111, 510–521. 10.1111/j.1471-4159.2009.06335.x 19682204 PMC2773444

[B21] Carliner H. Collins P. Y. Cabassa L. J. McNallen A. Joestl S. S. Lewis-Fernandez R. (2014). Prevalence of cardiovascular risk factors among racial and ethnic minorities with schizophrenia spectrum and bipolar disorders: a critical literature review. Compr. Psychiatry 55, 233–247. 10.1016/j.comppsych.2013.09.009 24269193 PMC4164219

[B22] Cascella N. G. Kryszak D. Bhatti B. Gregory P. Kelly D. L. Mc Evoy J. P.. (2009). Prevalence of celiac disease and gluten sensitivity in the United States clinical antipsychotic trials of intervention effectiveness study population. Schizophr. Bull. 37, 94–100. 10.1093/schbul/sbp055 19494248 PMC3004201

[B23] Cascella N. G. Santora D. Gregory P. Kelly D. L. Fasano A. Eaton W. W. (2013). Increased prevalence of transglutaminase 6 antibodies in sera from schizophrenia patients. Schizophr. Bull. 39, 867–871. 10.1093/schbul/sbs064 22516148 PMC3686447

[B24] Chambers R. A. Krystal J. H. Self D. W. (2001). A neurobiological basis for substance abuse comorbidity in schizophrenia. Biol. Psychiatry 50, 71–83. 10.1016/S0006-3223(01)01134-9 11526998 PMC2913410

[B25] Chaudhry I. B. Husain N. Drake R. Dunn G. Husain M. O. Kazmi A.. (2014). Add-on clinical effects of simvastatin and ondansetron in patients with schizophrenia stabilized on antipsychotic treatment: pilot study. Ther. Adv. Psychopharmacol. 4, 110–116. 10.1177/2045125313511487 25057343 PMC4107703

[B26] Chen A. T. Chibnall J. T. Nasrallah H. A. (2015). A meta-analysis of placebo-controlled trials of omega-3 fatty acid augmentation in schizophrenia: Possible stage-specific effects. Ann. Clin. Psychiatry 27, 289–296. 26554370

[B27] Chong H. Y. Teoh S. L. Wu D. B. Kotirum S. Chiou C. F. Chaiyakunapruk N. (2016). Global economic burden of schizophrenia: a systematic review. Neuropsychiatr. Dis. Treat. 12, 357–373. 10.2147/ndt.s96649 26937191 PMC4762470

[B28] Corlett P. R. Murray G. K. Honey G. D. Aitken M. R. Shanks D. R. Robbins T. W.. (2007). Disrupted prediction-error signal in psychosis: evidence for an associative account of delusions. Brain 130, 2387–2400. 10.1093/brain/awm173 17690132 PMC3838942

[B29] Correll C. U. Robinson D. G. Schooler N. R. Brunette M. F. Mueser K. T. Rosenheck R. A.. (2014). Cardiometabolic risk in patients with first-episode schizophrenia spectrum disorders: baseline results from the RAISE-ETP study. JAMA Psychiatry 71, 1350–1363. 10.1001/jamapsychiatry.2014.1314 25321337

[B30] Cummings J. H. Pomare E. W. Branch W. J. Naylor C. P. Macfarlane G. T. (1987). Short chain fatty acids in human large intestine, portal, hepatic and venous blood. Gut 28, 1221–1227. 10.1136/gut.28.10.1221 3678950 PMC1433442

[B31] Dealberto M. J. (2007). Why are immigrants at increased risk for psychosis? Vitamin D insufficiency, epigenetic mechanisms, or both? Med. Hypoth. 68, 259–267. 10.1016/j.mehy.2006.07.040 17011719

[B32] De Hert M. A. van Winkel R. Van Eyck D. Hanssens L. Wampers M. Scheen A.. (2006). Prevalence of the metabolic syndrome in patients with schizophrenia treated with antipsychotic medication. Schizophr. Res. 83, 87–93. 10.1016/j.schres.2005.12.855 16481149

[B33] De Hert M. Dockx L. Bernagie C. Peuskens B. Sweers K. Leucht S.. (2011). Prevalence and severity of antipsychotic related constipation in patients with schizophrenia: a retrospective descriptive study. BMC Gastroenterol. 11:17. 10.1186/1471-230X-11-17 21385443 PMC3062582

[B34] de Nijs J. Pet M. A. (2016). Metabolic syndrome in schizophrenia patients associated with poor premorbid school performance in early adolescence. Acta Psychiatr. Scand. 133, 289–297. 10.1111/acps.12528 26558719

[B35] De Palma G. Nadal I. Collado M. C. Sanz Y. (2009). Effects of a gluten-free diet on gut microbiota and immune function in healthy adult human subjects. Br. J. Nutr. 102, 1154–1160. 10.1017/S0007114509371767 19445821

[B36] De Santis A. Addolorato G. Romito A. Caputo S. Giordano A. Gambassi G.. (1997). Schizophrenic symptoms and SPECT abnormalities in a coeliac patient: regression after a gluten-free diet. J. Intern. Med. 242, 421–423. 10.1046/j.1365-2796.1997.00200.x 9408073

[B37] Dickerson F. Origoni A. Schroeder J. Schweinfurth L. A. Stallings C. Savage C. L.. (2016). Mortality in schizophrenia and bipolar disorder: clinical and serological predictors. Schizophr. Res. 170, 177–183. 10.1016/j.schres.2015.11.010 26607103

[B38] Dickerson F. Stallings C. Origoni A. Vaughan C. Khushalani S. Yang S.. (2013). C-reactive protein is elevated in schizophrenia. Schizophr. Res. 143, 198–202. 10.1016/j.schres.2012.10.041 23218564

[B39] Dieset I. Hope S. Ueland T. Bjella T. Agartz I. Melle I.. (2012). Cardiovascular risk factors during second generation antipsychotic treatment are associated with increased C-reactive protein. Schizophr. Res. 140, 169–174. 10.1016/j.schres.2012.06.040 22817875

[B40] Dimitrov D. H. Lee S. Yantis J. Honaker C. Braida N. Walss-Bass C. (2016). Elevated serum levels of high-sensitivity C-reactive proteins are associated with severe delusional symptoms in a subgroup of patients with schizophrenia. J. Clin. Psychiatry 77, 131–132. 10.4088/JCP.15l09833 26845270

[B41] Dixon L. Weiden P. Delahanty J. Goldberg R. Postrado L. Lucksted A.. (2000). Prevalence and correlates of diabetes in national schizophrenia samples. Schizophr. Bull. 26, 903–912. 10.1093/oxfordjournals.schbul.a033504 11087022

[B42] Dohan F. C. Grasberger J. C. (1973). Relapsed schizophrenics: earlier discharge from the hospital after cereal-free, milk-free diet. Am. J. Psychiatry 130, 685–688. 10.1176/ajp.130.6.685 4739849

[B43] Doorduin J. De Vries E. F. Willemsen A. T. De Groot J. C. Dierckx R. A. Klein H. C. (2009). Neuroinflammation in schizophrenia-related psychosis: a PET study. J. Nuclear Med. 50, 1801–1807. 10.2967/jnumed.109.066647 19837763

[B44] Dyall S. C. (2015). Long-chain omega-3 fatty acids and the brain: a review of the independent and shared effects of EPA, DPA and DHA. Front. Aging Neurosci. 7:52. 10.3389/fnagi.2015.00052 25954194 PMC4404917

[B45] Dyall S. C. Michael G. J. Whelpton R. Scott A. G. Michael-Titus A. T. (2007). Dietary enrichment with omega-3 polyunsaturated fatty acids reverses age-related decreases in the GluR2 and NR2B glutamate receptor subunits in rat forebrain. Neurobiol. Aging 28, 424–439. 10.1016/j.neurobiolaging.2006.01.002 16500747

[B46] Earls H. A. Curran T. Mittal V. (2016). A meta-analytic review of auditory event-related potential components as endophenotypes for schizophrenia: perspectives from first-degree relatives. Schizophr. Bull. 42, 1504–1516. 10.1093/schbul/sbw047 27217271 PMC5049529

[B47] Eaton W. W. Byrne M. Ewald H. Mors O. Chen C. Y. Agerbo E.. (2006). Association of schizophrenia and autoimmune diseases: linkage of Danish national registers. Am. J. Psychiatry 163, 521–528. 10.1176/appi.ajp.163.3.521 16513876

[B48] Elhayany A. Lustman A. Abel R. Attal-Singer J. Vinker S. (2010). A low carbohydrate Mediterranean diet improves cardiovascular risk factors and diabetes control among overweight patients with type 2 diabetes mellitus: a 1-year prospective randomized intervention study. Diabetes Obes. Metab. 12, 204–209. 10.1111/j.1463-1326.2009.01151.x 20151996

[B49] Elman I. Borsook D. Lukas S. E. (2006). Food intake and reward mechanisms in patients with schizophrenia: implications for metabolic disturbances and treatment with second-generation antipsychotic agents. Neuropsychopharmacology 31, 2091–2120. 10.1038/sj.npp.1301051 16541087

[B50] Emsley R. Myburgh C. Oosthuizen P. van Rensburg S. J. (2002). Randomized, placebo-controlled study of ethyl-eicosapentaenoic acid as supplemental treatment in schizophrenia. Am. J. Psychiatry 159, 1596–1598. 10.1176/appi.ajp.159.9.1596 12202284

[B51] Estruch R. Ros E. Salas-Salvado J. Covas M. I. Corella D. Aros F.. (2013). Primary prevention of cardiovascular disease with a Mediterranean diet. N. Engl. J. Med. 368, 1279–1290. 10.1056/NEJMoa1200303 23432189

[B52] Fan X. Goff D. C. Henderson D. C. (2007). Inflammation and schizophrenia. Expert Rev. Neurother. 7, 789–796. 10.1586/14737175.7.7.789 17610386

[B53] Fernandes B. S. Steiner J. Bernstein H. G. Dodd S. Pasco J. A. Dean O. M. (2016). C-reactive protein is increased in schizophrenia but is not altered by antipsychotics: meta-analysis and implications. Mol. Psychiatry 21, 554–564. 10.1038/mp.2015.87 26169974

[B54] Fernandez-Egea E. Bernardo M. Donner T. Conget I. Parellada E. Justicia A.. (2009). Metabolic profile of antipsychotic-naive individuals with non-affective psychosis. Br. J. Psychiatry 194, 434–438. 10.1192/bjp.bp.108.052605 19407273 PMC2703813

[B55] Fett A. K. Viechtbauer W. Dominguez M. D. Penn D. L. van Os J. Krabbendam L. (2011). The relationship between neurocognition and social cognition with functional outcomes in schizophrenia: a meta-analysis. Neurosci. Biobehav. Rev. 35, 573–588. 10.1016/j.neubiorev.2010.07.001 20620163

[B56] Feyh A. Bracero L. Lakhani H. V. Santhanam P. Shapiro J. I. Khitan Z.. (2016). Role of dietary components in modulating hypertension. J. Clin. Exper. Cardiol. 7:433. 10.4172/2155-9880.1000433 27158555 PMC4857880

[B57] Fioravanti M. Carlone O. Vitale B. Cinti M. E. Clare L. (2005). A meta-analysis of cognitive deficits in adults with a diagnosis of schizophrenia. Neuropsychol. Rev. 15, 73–95. 10.1007/s11065-005-6254-9 16211467

[B58] Foster G. D. Wyatt H. R. Hill J. O. McGuckin B. G. Brill C. Mohammed B. S.. (2003). A randomized trial of a low-carbohydrate diet for obesity. N. Engl. J. Med. 348, 2082–2090. 10.1056/NEJMoa022207 12761365

[B59] Gao X. M. Sakai K. Roberts R. C. Conley R. R. Dean B. Tamminga C. A. (2000). Ionotropic glutamate receptors and expression of N-methyl-D-aspartate receptor subunits in subregions of human hippocampus: effects of schizophrenia. Am. J. Psychiatry 157, 1141–1149. 10.1176/appi.ajp.157.7.1141 10873924

[B60] Garrido-Mesa N. Zarzuelo A. Galvez J. (2013). What is behind the non-antibiotic properties of minocycline? Pharmacol. Res. 67, 18–30. 10.1016/j.phrs.2012.10.006 23085382

[B61] Goff D. C. Sullivan L. M. McEvoy J. P. Meyer J. M. Nasrallah H. A. Daumit G. L.. (2005). A comparison of ten-year cardiac risk estimates in schizophrenia patients from the CATIE study and matched controls. Schizophr. Res. 80, 45–53. 10.1016/j.schres.2005.08.010 16198088

[B62] Gold J. M. Carpenter C. Randolph C. Goldberg T. E. Weinberger D. R. (1997). Auditory working memory and Wisconsin Card Sorting Test performance in schizophrenia. Arch. Gen. Psychiatry 54, 159–165. 10.1001/archpsyc.1997.01830140071013 9040284

[B63] Gold J. M. Waltz J. A. Matveeva T. M. Kasanova Z. Strauss G. P. Herbener E. S.. (2012). Negative symptoms and the failure to represent the expected reward value of actions: behavioral and computational modeling evidence. Arch. Gen. Psychiatry 69, 129–138. 10.1001/archgenpsychiatry.2011.1269 22310503 PMC4406055

[B64] Goldman-Rakic P. S. (1994). Working memory dysfunction in schizophrenia. J. Neuropsychiatry Clin. Neurosci. 6, 348–357. 10.1176/jnp.6.4.348 7841806

[B65] Goughari A. S. Mazhari S. Pourrahimi A. M. Sadeghi M. M. Nakhaee N. (2015). Associations between components of metabolic syndrome and cognition in patients with schizophrenia. J. Psychiatr. Pract. 21, 190–197. 10.1097/PRA.0000000000000065 25955261

[B66] Grunder G. Heinze M. Cordes J. Muhlbauer B. Juckel G. Schulz C.. (2016). Effects of first-generation antipsychotics versus second-generation antipsychotics on quality of life in schizophrenia: a double-blind, randomised study. Lancet Psychiatry 3, 717–729. 10.1016/s2215-0366(16)00085-7 27265548

[B67] Gur R. E. Gur R. C. Skolnick B. E. Caroff S. Obrist W. D. Resnick S.. (1985). Brain function in psychiatric disorders: III. Regional cerebral blood flow in unmedicated schizophrenics. Arch. Gen. Psychiatry 42, 329–334. 10.1001/archpsyc.1985.01790270015001 2858190

[B68] Gutierrez-Diaz I. Fernandez-Navarro T. Sanchez B. Margolles A. Gonzalez S. (2016). Mediterranean diet and faecal microbiota: a transversal study. Food Funct. 7, 2347–2356. 10.1039/C6FO00105J 27137178

[B69] Harris W. S. Miller M. Tighe A. P. Davidson M. H. Schaefer E. J. (2008). Omega-3 fatty acids and coronary heart disease risk: clinical and mechanistic perspectives. Atherosclerosis 197, 12–24. 10.1016/j.atherosclerosis.2007.11.008 18160071

[B70] Hazlett E. A. Rothstein E. G. Ferreira R. Silverman J. M. Siever L. J. Olincy A. (2015). Sensory gating disturbances in the spectrum: similarities and differences in schizotypal personality disorder and schizophrenia. Schizophr. Res. 161, 283–290. 10.1016/j.schres.2014.11.020 25482574 PMC4308515

[B71] Heald A. Sein K. Anderson S. Pendlebury J. Guy M. Narayan V.. (2015). Diet, exercise and the metabolic syndrome in schizophrenia: a cross-sectional study. Schizophr. Res. 169, 494–495. 10.1016/j.schres.2015.10.043 26589389

[B72] Hemmings G. (2004). Schizophrenia. Lancet 364, 1312–1313. 10.1016/s0140-6736(04)17181-x 15474128

[B73] Hemmings G. P. (1990). Is schizophrenia a G-I disease. Br. J. Psychiatry 156, 448. 10.1192/bjp.156.3.448b 2346859

[B74] Henderson D. C. Borba C. P. Daley T. B. Boxill R. Nguyen D. D. Culhane M. A.. (2006). Dietary intake profile of patients with schizophrenia. Ann. Clin. Psychiatry 18, 99–105. 10.1080/10401230600614538 16754415

[B75] Hennekens C. H. Hennekens A. R. Hollar D. Casey D. E. (2005). Schizophrenia and increased risks of cardiovascular disease. Am. Heart J. 150, 1115–1121. 10.1016/j.ahj.2005.02.007 16338246

[B76] Ho B.-C. Andreasen N. C. Nopoulos P. Arndt S. Magnotta V. Flaum M. (2003). Progressive structural brain abnormalities and their relationship to clinical outcome: a longitudinal magnetic resonance imaging study early in schizophrenia. Arch. Gen. Psychiatry 60, 585–594. 10.1001/archpsyc.60.6.585 12796222

[B77] Hoffer L. J. (2008). Vitamin therapy in schizophrenia. Isr. J. Psychiatry Relat. Sci. 45, 3–10. 18587164

[B78] Hong H. Kim B. S. Im H. I. (2016). Pathophysiological role of neuroinflammation in neurodegenerative diseases and psychiatric disorders. Int. Neurourol. J. 20, S2–S7. 10.5213/inj.1632604.302 27230456 PMC4895907

[B79] Hotamisligil G. S. (2006). Inflammation and metabolic disorders. Nature 444, 860–867. 10.1038/nature05485 17167474

[B80] Howes O. D. Kapur S. (2009). The dopamine hypothesis of schizophrenia: version III—the final common pathway. Schizophr. Bull. 35, 549–562. 10.1093/schbul/sbp006 19325164 PMC2669582

[B81] Hsiao C. C. Ree S. C. Chiang Y. L. Yeh S. S. Chen C. K. (2004). Obesity in schizophrenic outpatients receiving antipsychotics in Taiwan. Psychiatry Clin. Neurosci. 58, 403–409. 10.1111/j.1440-1819.2004.01275.x 15298654

[B82] Hudson Z. D. Miller B. J. (2016). Meta-analysis of cytokine and chemokine genes in schizophrenia. Clin. Schizophr. Relat. Psychoses. [Epub ahead of print]. 10.3371/CSRP.HUMI.070516 27454212

[B83] Hutton S. B. Puri B. K. Duncan L. J. Robbins T. W. Barnes T. R. Joyce E. M. (1998). Executive function in first-episode schizophrenia. Psychol. Med. 28, 463–473. 10.1017/S0033291797006041 9572103

[B84] Huuskonen J. Suuronen T. Nuutinen T. Kyrylenko S. Salminen A. (2004). Regulation of microglial inflammatory response by sodium butyrate and short-chain fatty acids. Br. J. Pharmacol. 141, 874–880. 10.1038/sj.bjp.0705682 14744800 PMC1574260

[B85] Innis S. M. (2008). Dietary omega 3 fatty acids and the developing brain. Brain Res. 1237, 35–43. 10.1016/j.brainres.2008.08.078 18789910

[B86] Inta D. Lang U. E. Borgwardt S. Meyer-Lindenberg A. Gass P. (2016). Microglia activation and schizophrenia: lessons from the effects of minocycline on postnatal neurogenesis, neuronal survival and synaptic pruning. Schizophr. Bull. [Epub ahead of print]. 10.1093/schbul/sbw088 27352782 PMC5464012

[B87] Ito H. Kumagai T. Kimura M. Koike S. Shimizu T. (2015). Dietary intake in body mass index differences in community-based Japanese patients with Schizophrenia. Iran. J. Public Health 44, 639–645. 26284204 PMC4537620

[B88] Itoh Y. Kawamata Y. Harada M. Kobayashi M. Fujii R. Fukusumi S.. (2003). Free fatty acids regulate insulin secretion from pancreatic β cells through GPR40. Nature 422, 173–176. 10.1038/nature01478 12629551

[B89] Jackson J. Eaton W. Cascella N. Fasano A. Santora D. Sullivan K.. (2014). Gluten sensitivity and relationship to psychiatric symptoms in people with schizophrenia. Schizophr. Res. 159, 539–542. 10.1016/j.schres.2014.09.023 25311778 PMC4476307

[B90] Jackson J. R. Eaton W. W. Cascella N. G. Fasano A. Kelly D. L. (2012). Neurologic and psychiatric manifestations of celiac disease and gluten sensitivity. Psychiatr. Q. 83, 91–102. 10.1007/s11126-011-9186-y 21877216 PMC3641836

[B91] Jarskog L. F. Hamer R. M. Catellier D. J. Stewart D. D. Lavange L. Ray N.. (2013). Metformin for weight loss and metabolic control in overweight outpatients with schizophrenia and schizoaffective disorder. Am. J. Psychiatry 170, 1032–1040. 10.1176/appi.ajp.2013.12010127 23846733 PMC3874085

[B92] Ji E. Weickert C. S. Lenroot R. Kindler J. Skilleter A. J. Vercammen A.. (2016). Adjunctive selective estrogen receptor modulator increases neural activity in the hippocampus and inferior frontal gyrus during emotional face recognition in schizophrenia. Transl. Psychiatry 6:e795. 10.1038/tp.2016.59 27138794 PMC5070055

[B93] Jin H. Folsom D. Sasaki A. Mudaliar S. Henry R. Torres M.. (2011). Increased Framingham 10-year risk of coronary heart disease in middle-aged and older patients with psychotic symptoms. Schizophr. Res. 125, 295–299. 10.1016/j.schres.2010.10.029 21093219 PMC3031775

[B94] Jones P. B. Bebbington P. Foerster A. Lewis S. W. Murray R. M. Russell A.. (1993). Premorbid social underachievement in schizophrenia. Results from the Camberwell Collaborative Psychosis Study. Br. J. Psychiatry 162, 65–71. 10.1192/bjp.162.1.65 8425142

[B95] Joseph J. Depp C. Martin A. S. Daly R. E. Glorioso D. K. Palmer B. W.. (2015). Associations of high sensitivity C-reactive protein levels in schizophrenia and comparison groups. Schizophr. Res. 168, 456–460. 10.1016/j.schres.2015.08.019 26341579 PMC4591213

[B96] Juckel G. Schlagenhauf F. Koslowski M. Wustenberg T. Villringer A. Knutson B.. (2006). Dysfunction of ventral striatal reward prediction in schizophrenia. NeuroImage 29, 409–416. 10.1016/j.neuroimage.2005.07.051 16139525

[B97] Judge M. P. Casavant S. G. Dias J. A. McGrath J. M. (2016). Reduced DHA transfer in diabetic pregnancies: mechanistic basis and long-term neurodevelopmental implications. Nutr. Rev. 74, 411–420. 10.1093/nutrit/nuw006 27142302 PMC4892301

[B98] Kalaydjian A. E. Eaton W. Cascella N. Fasano A. (2006). The gluten connection: the association between schizophrenia and celiac disease. Acta Psychiatr. Scand. 113, 82–90. 10.1111/j.1600-0447.2005.00687.x 16423158

[B99] Kale A. Naphade N. Sapkale S. Kamaraju M. Pillai A. Joshi S.. (2010). Reduced folic acid, vitamin B12 and docosahexaenoic acid and increased homocysteine and cortisol in never-medicated schizophrenia patients: implications for altered one-carbon metabolism. Psychiatry Res. 175, 47–53. 10.1016/j.psychres.2009.01.013 19969375

[B100] Kapur S. (2003). Psychosis as a state of aberrant salience: a framework linking biology, phenomenology, and pharmacology in schizophrenia. Am. J. Psychiatry 160, 13–23. 10.1176/appi.ajp.160.1.13 12505794

[B101] Kianimehr G. Fatehi F. Hashempoor S. Khodaei-Ardakani M. R. Rezaei F. Nazari A.. (2014). Raloxifene adjunctive therapy for postmenopausal women suffering from chronic schizophrenia: a randomized double-blind and placebo controlled trial. Daru 22:55. 10.1186/2008-2231-22-55 25012765 PMC4100751

[B102] Kim C. H. Park J. Kim M. (2014). Gut microbiota-derived short-chain Fatty acids, T cells, and inflammation. Immune Netw. 14, 277–288. 10.4110/in.2014.14.6.277 25550694 PMC4275385

[B103] Kinsman S. L. Vining E. P. Quaskey S. A. Mellits D. Freeman J. M. (1992). Efficacy of the ketogenic diet for intractable seizure disorders: review of 58 cases. Epilepsia 33, 1132–1136. 10.1111/j.1528-1157.1992.tb01770.x 1464275

[B104] Koloverou E. Panagiotakos D. B. Pitsavos C. Chrysohoou C. Georgousopoulou E. N. Grekas A.. (2016). Adherence to Mediterranean diet and 10-year incidence (2002-2012) of diabetes: correlations with inflammatory and oxidative stress biomarkers in the ATTICA cohort study. Diabetes Metab. Res. Rev. 32, 73–81. 10.1002/dmrr.2672 26104243

[B105] Kou W. Luchtman D. Song C. (2008). Eicosapentaenoic acid (EPA) increases cell viability and expression of neurotrophin receptors in retinoic acid and brain-derived neurotrophic factor differentiated SH-SY5Y cells. Eur. J. Nutr. 47, 104–113. 10.1007/s00394-008-0703-1 18360785

[B106] Kraeuter A. K. Loxton H. Lima B. C. Rudd D. Sarnyai Z. (2015). Ketogenic diet reverses behavioral abnormalities in an acute NMDA receptor hypofunction model of schizophrenia. Schizophr. Res. 169, 491–493. 10.1016/j.schres.2015.10.041 26547882

[B107] Kraft B. D. Westman E. C. (2009). Schizophrenia, gluten, and low-carbohydrate, ketogenic diets: a case report and review of the literature. Nutr. Metab. (Lond). 6:10. 10.1186/1743-7075-6-10 19245705 PMC2652467

[B108] Kulkarni J. Gavrilidis E. Gwini S. M. Worsley R. Grigg J. Warren A.. (2016). Effect of adjunctive raloxifene therapy on severity of refractory Schizophrenia in women: a randomized clinical trial. JAMA Psychiatry 73, 947–954. 10.1001/jamapsychiatry.2016.1383 27438995

[B109] Kwiterovich P. O. Jr. Vining E. P. Pyzik P. Skolasky R. Jr. Freeman J. M. (2003). Effect of a high-fat ketogenic diet on plasma levels of lipids, lipoproteins, and apolipoproteins in children. JAMA 290, 912–920. 10.1001/jama.290.7.912 12928468

[B110] Lakoski S. G. Greenland P. Wong N. D. Schreiner P. J. Herrington D. M. Kronmal R. A.. (2007). Coronary artery calcium scores and risk for cardiovascular events in women classified as “low risk” based on Framingham risk score: the multi-ethnic study of atherosclerosis (MESA). Arch. Intern. Med. 167, 2437–2442. 10.1001/archinte.167.22.2437 18071165

[B111] Larsson S. C. Wallin A. Wolk A. (2016). Dietary approaches to stop hypertension diet and incidence of stroke: results from 2 prospective cohorts. Stroke 47, 986–990. 10.1161/STROKEAHA.116.012675 26869384

[B112] Le Poul E. Loison C. Struyf S. Springael J. Y. Lannoy V. Decobecq M. E.. (2003). Functional characterization of human receptors for short chain fatty acids and their role in polymorphonuclear cell activation. J. Biol. Chem. 278, 25481–25489. 10.1074/jbc.M301403200 12711604

[B113] Lewis D. A. Levitt P. (2002). Schizophrenia as a disorder of neurodevelopment. Annu. Rev. Neurosci. 25, 409–432. 10.1146/annurev.neuro.25.112701.142754 12052915

[B114] Li H. P. Chen X. Li M. Q. (2013). Butyrate alleviates metabolic impairments and protects pancreatic β cell function in pregnant mice with obesity. Int. J. Clin. Exp. Pathol. 6, 1574–1584. 23923076 PMC3726973

[B115] Lindenmayer J. P. Khan A. Kaushik S. Thanju A. Praveen R. Hoffman L.. (2012). Relationship between metabolic syndrome and cognition in patients with schizophrenia. Schizophr. Res. 142, 171–176. 10.1016/j.schres.2012.09.019 23106932

[B116] Lionetti E. Leonardi S. Franzonello C. Mancardi M. Ruggieri M. Catassi C. (2015). Gluten psychosis: confirmation of a new clinical entity. Nutrients 7, 5532–5539. 10.3390/nu7075235 26184290 PMC4517012

[B117] Ma Y. Griffith J. A. Chasan-Taber L. Olendzki B. C. Jackson E. Stanek E. J. III. (2006). Association between dietary fiber and serum C-reactive protein. Am. J. Clin. Nutr. 83, 760–766. 16600925 10.1093/ajcn/83.4.760 PMC1456807

[B118] Manoach D. S. (2003). Prefrontal cortex dysfunction during working memory performance in schizophrenia: reconciling discrepant findings. Schizophr. Res. 60, 285–298. 10.1016/S0920-9964(02)00294-3 12591590

[B119] Mathew A. V. Seymour E. M. Byun J. Pennathur S. Hummel S. L. (2015). Altered metabolic profile with sodium-restricted dietary approaches to stop hypertension diet in hypertensive heart failure with preserved ejection fraction. J. Card. Fail. 21, 963–967. 10.1016/j.cardfail.2015.10.003 26497755 PMC4720261

[B120] McCreadie R. G. (2003). Diet, smoking and cardiovascular risk in people with schizophrenia: descriptive study. Br. J. Psychiatry 183, 534–539. 10.1192/bjp.183.6.534 14645025

[B121] McCreadie R. Macdonald E. Blacklock C. Tilak-Singh D. Wiles D. Halliday J.. (1998). Dietary intake of schizophrenic patients in Nithsdale, Scotland: case-control study. BMJ 317, 784–785. 10.1136/bmj.317.7161.784 9740565 PMC28668

[B122] McEvoy J. P. Meyer J. M. Goff D. C. Nasrallah H. A. Davis S. M. Sullivan L.. (2005). Prevalence of the metabolic syndrome in patients with schizophrenia: baseline results from the Clinical Antipsychotic Trials of Intervention Effectiveness (CATIE) schizophrenia trial and comparison with national estimates from NHANES III. Schizophr. Res. 80, 19–32. 10.1016/j.schres.2005.07.014 16137860

[B123] McNamara R. K. Jandacek R. Rider T. Tso P. Hahn C. G. Richtand N. M.. (2007). Abnormalities in the fatty acid composition of the postmortem orbitofrontal cortex of schizophrenic patients: gender differences and partial normalization with antipsychotic medications. Schizophr. Res. 91, 37–50. 10.1016/j.schres.2006.11.027 17236749 PMC1853256

[B124] Meijer K. de Vos P. Priebe M. G. (2010). Butyrate and other short-chain fatty acids as modulators of immunity: what relevance for health? Curr. Opin. Clin. Nutr. Metab. Care 13, 715–721. 10.1097/MCO.0b013e32833eebe5 20823773

[B125] Meyer U. (2013). Developmental neuroinflammation and schizophrenia. Progr. Neuro Psychopharmacol. Biol. Psychiatry 42, 20–34. 10.1016/j.pnpbp.2011.11.003 22122877

[B126] Micoulaud-Franchi J. A. Vaillant F. Lopez R. Peri P. Baillif A. Brandejsky L.. (2015). Sensory gating in adult with attention-deficit/hyperactivity disorder: event-evoked potential and perceptual experience reports comparisons with schizophrenia. Biol. Psychol. 107, 16–23. 10.1016/j.biopsycho.2015.03.002 25766264

[B127] Miller B. J. Buckley P. Seabolt W. Mellor A. Kirkpatrick B. (2011). Meta-analysis of cytokine alterations in schizophrenia: clinical status and antipsychotic effects. Biol. Psychiatry 70, 663–671. 10.1016/j.biopsych.2011.04.013 21641581 PMC4071300

[B128] Miller B. J. Culpepper N. Rapaport M. H. (2014). C-reactive protein levels in schizophrenia: a review and meta-analysis. Clin. Schizophr. Relat. Psychoses 7, 223–230. 10.3371/CSRP.MICU.020813 23428789

[B129] Miller E. R. III Erlinger T. P. Young D. R. Jehn M. Charleston J. Rhodes D.. (2002). Results of the Diet, Exercise, and Weight Loss Intervention Trial (DEW-IT). Hypertension 40, 612–618. 10.1161/01.HYP.0000037217.96002.8E 12411452

[B130] Mitchell A. J. Vancampfort D. Sweers K. van Winkel R. Yu W. De Hert M. (2013). Prevalence of metabolic syndrome and metabolic abnormalities in schizophrenia and related disorders–a systematic review and meta-analysis. Schizophr. Bull. 39, 306–318. 10.1093/schbul/sbr148 22207632 PMC3576174

[B131] Monji A. Kato T. A. Mizoguchi Y. Horikawa H. Seki Y. Kasai M.. (2013). Neuroinflammation in schizophrenia especially focused on the role of microglia. Prog. Neuropsychopharmacol. Biol. Psychiatry 42, 115–121. 10.1016/j.pnpbp.2011.12.002 22192886

[B132] Morris R. Griffiths O. Le Pelley M. E. Weickert T. W. (2012). Attention to irrelevant cues is related to positive symptoms in schizophrenia. Schizophr. Bull. 39, 575–582. 10.1093/schbul/sbr192 22267535 PMC3627774

[B133] Mozaffarian D. Wu J. H. (2011). Omega-3 fatty acids and cardiovascular disease: effects on risk factors, molecular pathways, and clinical events. J. Am. Coll. Cardiol. 58, 2047–2067. 10.1016/j.jacc.2011.06.063 22051327

[B134] Muller N. Weidinger E. Leitner B. Schwarz M. J. (2015). The role of inflammation in schizophrenia. Front. Neurosci. 9:372. 10.3389/fnins.2015.00372 26539073 PMC4612505

[B135] Murray J. A. (1999). The widening spectrum of celiac disease. Am. J. Clin. Nutr. 69, 354–365. 10075317 10.1093/ajcn/69.3.354

[B136] Na K. S. Jung H. Y. Kim Y. K. (2014). The role of pro-inflammatory cytokines in the neuroinflammation and neurogenesis of schizophrenia. Prog. Neuropsychopharmacol. Biol. Psychiatry 48, 277–286. 10.1016/j.pnpbp.2012.10.022 23123365

[B137] Nasrallah H. A. Meyer J. M. Goff D. C. McEvoy J. P. Davis S. M. Stroup T. S.. (2006). Low rates of treatment for hypertension, dyslipidemia and diabetes in schizophrenia: data from the CATIE schizophrenia trial sample at baseline. Schizophr. Res. 86, 15–22. 10.1016/j.schres.2006.06.026 16884895

[B138] Neale E. P. Batterham M. J. Tapsell L. C. (2016). Consumption of a healthy dietary pattern results in significant reductions in C-reactive protein levels in adults: a meta-analysis. Nutr. Res. 36, 391–401. 10.1016/j.nutres.2016.02.009 27101757

[B139] Nilsson N. E. Kotarsky K. Owman C. Olde B. (2003). Identification of a free fatty acid receptor, FFA2R, expressed on leukocytes and activated by short-chain fatty acids. Biochem. Biophys. Res. Commun. 303, 1047–1052. 10.1016/S0006-291X(03)00488-1 12684041

[B140] Niswander G. D. Haslerud G. M. Mitchell G. D. (1963). Effect of catatonia on schizophrenic mortality. Arch. Gen. Psychiatry 9, 548–551. 10.1001/archpsyc.1963.01720180020003 14056835

[B141] Nohr M. K. Egerod K. L. Christiansen S. H. Gille A. Offermanns S. Schwartz T. W.. (2015). Expression of the short chain fatty acid receptor GPR41/FFAR3 in autonomic and somatic sensory ganglia. Neuroscience 290, 126–137. 10.1016/j.neuroscience.2015.01.040 25637492

[B142] Norden D. M. Muccigrosso M. M. Godbout J. P. (2015). Microglial priming and enhanced reactivity to secondary insult in aging, and traumatic CNS injury, and neurodegenerative disease. Neuropharmacology 96, 29–41. 10.1016/j.neuropharm.2014.10.028 25445485 PMC4430467

[B143] Norden D. M. Trojanowski P. J. Villanueva E. Navarro E. Godbout J. P. (2016). Sequential activation of microglia and astrocyte cytokine expression precedes increased iba-1 or GFAP immunoreactivity following systemic immune challenge. Glia 64, 300–316. 10.1002/glia.22930 26470014 PMC4707977

[B144] Norlelawati A. T. Kartini A. Ramli M. Norsidah K. Wan Azizi W. S. Tariq A. R. (2012). Obesity in multiracial schizophrenia patients receiving outpatient treatment in a regional tertiary hospital in malaysia. East Asian Arch. Psychiatry 22, 49–56. 22714874

[B145] Nsaiba M. J. Lapointe M. Mabrouk H. Douki W. Gaha L. Perusse L.. (2015). C3 polymorphism influences circulating levels of C3, ASP and lipids in schizophrenic patients. Neurochem. Res. 40, 906–914. 10.1007/s11064-015-1543-z 25720829

[B146] Nuechterlein K. H. Barch D. M. Gold J. M. Goldberg T. E. Green M. F. Heaton R. K. (2004). Identification of separable cognitive factors in schizophrenia. Schizophr. Res. 72, 29–39. 10.1016/j.schres.2004.09.007 15531405

[B147] Nunes D. Eskinazi B. Camboim Rockett F. Delgado V. B. Schweigert Perry I. D. (2014). Nutritional status, food intake and cardiovascular disease risk in individuals with schizophrenia in southern Brazil: a case-control study. Rev. Psiquiatr. Salud Ment. 7, 72–79. 10.1016/j.rpsm.2013.07.001 24054065

[B148] Oh D. Y. Talukdar S. Bae E. J. Imamura T. Morinaga H. Fan W.. (2010). GPR120 is an omega-3 fatty acid receptor mediating potent anti-inflammatory and insulin-sensitizing effects. Cell 142, 687–698. 10.1016/j.cell.2010.07.041 20813258 PMC2956412

[B149] Okusaga O. Yolken R. H. Langenberg P. Sleemi A. Kelly D. L. Vaswani D.. (2013). Elevated gliadin antibody levels in individuals with schizophrenia. World J. Biol. Psychiatry 14, 509–515. 10.3109/15622975.2012.747699 23282016

[B150] Oldendorf W. H. (1973). Carrier-mediated blood-brain barrier transport of short-chain monocarboxylic organic acids. Am. J. Physiol. Legacy Content 224, 1450–1453. 4712154 10.1152/ajplegacy.1973.224.6.1450

[B151] Olfson M. Gerhard T. Huang C. Crystal S. Stroup T. S. (2015). Premature mortality among adults with schizophrenia in the United States. JAMA Psychiatry 72, 1172–1181. 10.1001/jamapsychiatry.2015.1737 26509694

[B152] Ozmen B. Kirmaz C. Aydin K. Kafesciler S. O. Guclu F. Hekimsoy Z. (2007). Influence of the selective oestrogen receptor modulator (raloxifene hydrochloride) on IL-6, TNF-alpha, TGF-beta1 and bone turnover markers in the treatment of postmenopausal osteoporosis. Eur. Cytokine Netw. 18, 148–153. 17823083 10.1684/ecn.2007.0097

[B153] Pacheco A. Easterling W. S. Pryer M. W. (1965). A pilot study of the ketogenic diet in schizophrenia. Am. J. Psychiatry 121, 1110–1111. 10.1176/ajp.121.11.1110 14283310

[B154] Peet M. (2003). Nutrition and schizophrenia: an epidemiological and clinical perspective. Nutr. Health 17, 211–219. 10.1177/026010600301700304 14703154

[B155] Peet M. (2004). Diet, diabetes and schizophrenia: review and hypothesis. Br. J. Psychiatry 184, s102–s105. 10.1192/bjp.184.47.s102 15056602

[B156] Peet M. (2006). The metabolic syndrome, omega-3 fatty acids and inflammatory processes in relation to schizophrenia. Prostaglandins Leukot. Essent. Fatty Acids 75, 323–327. 10.1016/j.plefa.2006.07.013 16934964

[B157] Peet M. Brind J. Ramchand C. N. Shah S. Vankar G. K. (2001). Two double-blind placebo-controlled pilot studies of eicosapentaenoic acid in the treatment of schizophrenia. Schizophr. Res. 49, 243–251. 10.1016/S0920-9964(00)00083-9 11356585

[B158] Penn A. H. Schmid-Schonbein G. W. (2008). The intestine as source of cytotoxic mediators in shock: free fatty acids and degradation of lipid-binding proteins. Am. J. Physiol. Heart Circ. Physiol. 294, H1779–H1792. 10.1152/ajpheart.00902.2007 18263716

[B159] Pillinger T. Beck K. Gobjila C. Donocik J. G. Jauhar S. Howes O. D. (2017). Impaired glucose homeostasis in first-episode schizophrenia: a systematic review and meta-analysis. JAMA Psychiatry 74, 261–269. 10.1001/jamapsychiatry.2016.3803 28097367 PMC6352957

[B160] Prasad K. (2006). C-reactive protein (CRP)-lowering agents. Cardiovasc. Drug Rev. 24, 33–50. 10.1111/j.1527-3466.2006.00033.x 16939632

[B161] Puglisi M. J. Fernandez M. L. (2008). Modulation of C-reactive protein, tumor necrosis factor-alpha, and adiponectin by diet, exercise, and weight loss. J. Nutr. 138, 2293–2296. 10.3945/jn.108.097188 19022947

[B162] Rabinowitz J. Levine S. Z. Garibaldi G. Bugarski-Kirola D. Berardo C. G. Kapur S. (2012). Negative symptoms have greater impact on functioning than positive symptoms in schizophrenia: analysis of CATIE data. Schizophr. Res. 137, 147–150. 10.1016/j.schres.2012.01.015 22316568

[B163] Rahman M. Muhammad S. Khan M. A. Chen H. Ridder D. A. Muller-Fielitz H.. (2014). The beta-hydroxybutyrate receptor HCA2 activates a neuroprotective subset of macrophages. Nat. Commun. 5, 3944. 10.1038/ncomms4944 24845831

[B164] Rao J. S. Kim H. W. Harry G. J. Rapoport S. I. Reese E. A. (2013). Increased neuroinflammatory and arachidonic acid cascade markers, and reduced synaptic proteins, in the postmortem frontal cortex from schizophrenia patients. Schizophr. Res. 147, 24–31. 10.1016/j.schres.2013.02.017 23566496 PMC3812915

[B165] Ridker P. M. (2001). High-sensitivity C-reactive protein: potential adjunct for global risk assessment in the primary prevention of cardiovascular disease. Circulation 103, 1813–1818. 10.1161/01.CIR.103.13.1813 11282915

[B166] Ripke S. Neale B. M. Corvin A. Walters J. T. Farh K. H. Holmans P. A.. (2014). Biological insights from 108 schizophrenia-associated genetic loci. Nature 511, 421. 10.1038/nature13595 25056061 PMC4112379

[B167] Roberfroid M. Gibson G. R. Hoyles L. McCartney A. L. Rastall R. Rowland I.. (2010). Prebiotic effects: metabolic and health benefits. Br. J. Nutr. 104 (Suppl. 2), S1–S63. 10.1017/s0007114510003363 20920376

[B168] Rodriguez-Sanchez J. M. Crespo-Facorro B. Gonzalez-Blanch C. Perez-Iglesias R. Vazquez-Barquero J. L. (2007). Cognitive dysfunction in first-episode psychosis: the processing speed hypothesis. Br. J. Psychiatry Suppl. 51, s107–s110. 10.1192/bjp.191.51.s107 18055925

[B169] Roth T. L. Lubin F. D. Sodhi M. Kleinman J. E. (2009). Epigenetic mechanisms in schizophrenia. Biochim. Biophys. Acta 1790, 869–877. 10.1016/j.bbagen.2009.06.009 19559755 PMC2779706

[B170] Ryan M. C. Collins P. Thakore J. H. (2003). Impaired fasting glucose tolerance in first-episode, drug-naive patients with schizophrenia. Am. J. Psychiatry 160, 284–289. 10.1176/appi.ajp.160.2.284 12562574

[B171] Sabbag S. Twamley E. W. Vella L. Heaton R. K. Patterson T. L. Harvey P. D. (2012). Predictors of the accuracy of self assessment of everyday functioning in people with schizophrenia. Schizophr. Res. 137, 190–195. 10.1016/j.schres.2012.02.002 22386735 PMC3351672

[B172] Sacks F. M. Svetkey L. P. Vollmer W. M. Appel L. J. Bray G. A. Harsha D.. (2001). Effects on blood pressure of reduced dietary sodium and the Dietary Approaches to Stop Hypertension (DASH) diet. DASH-Sodium Collaborative Research Group. New Engl. J. Med. 344, 3–10. 10.1056/nejm200101043440101 11136953

[B173] Saha S. Chant D. McGrath J. (2007). A systematic review of mortality in schizophrenia: is the differential mortality gap worsening over time? Arch. Gen. Psychiatry 64, 1123–1131. 10.1001/archpsyc.64.10.1123 17909124

[B174] Schenkel L. S. Spaulding W. D. Silverstein S. M. (2005). Poor premorbid social functioning and theory of mind deficit in schizophrenia: evidence of reduced context processing? J. Psychiatr. Res. 39, 499–508. 10.1016/j.jpsychires.2005.01.001 15992559

[B175] Schmid-Schonbein G. W. (2006). Analysis of inflammation. Annu. Rev. Biomed. Eng. 8, 93–131. 10.1146/annurev.bioeng.8.061505.095708 16834553

[B176] Seeman M. V. (2007). An outcome measure in schizophrenia: mortality. Can. J. Psychiatry 52, 55–60. 10.1177/070674370705200109 17444079

[B177] Sekar A. Bialas A. R. de Rivera H. Davis A. Hammond T. R. Kamitaki N.. (2016). Schizophrenia risk from complex variation of complement component 4. Nature 530, 177–183. 10.1038/nature16549 26814963 PMC4752392

[B178] Selkrig J. Wong P. Zhang X. Pettersson S. (2014). Metabolic tinkering by the gut microbiome: implications for brain development and function. Gut. Microb. 5, 369–380. 10.4161/gmic.28681 24685620 PMC4153776

[B179] Severance E. G. Gressitt K. L. Halling M. Stallings C. R. Origoni A. E. Vaughan C.. (2012). Complement C1q formation of immune complexes with milk caseins and wheat glutens in schizophrenia. Neurobiol. Dis. 48, 447–453. 10.1016/j.nbd.2012.07.005 22801085 PMC3465075

[B180] Severance E. G. Gressitt K. L. Stallings C. R. Katsafanas E. Schweinfurth L. A. Savage C. L.. (2016a). *Candida albicans* exposures, sex specificity and cognitive deficits in schizophrenia and bipolar disorder. NPJ Schizophr. 2, 16018. 10.1038/npjschz.2016.18 27336058 PMC4898895

[B181] Severance E. G. Gressitt K. L. Stallings C. R. Origoni A. E. Khushalani S. Leweke F. M.. (2013). Discordant patterns of bacterial translocation markers and implications for innate immune imbalances in schizophrenia. Schizophr. Res. 148, 130–137. 10.1016/j.schres.2013.05.018 23746484 PMC3732507

[B182] Severance E. G. Xiao J. Jones-Brando L. Sabunciyan S. Li Y. Pletnikov M.. (2016b). *Toxoplasma gondii*-A Gastrointestinal Pathogen Associated with Human Brain Diseases. Int. Rev. Neurobiol. 131, 143–163. 10.1016/bs.irn.2016.08.008 27793216 PMC5508738

[B183] Shai I. Schwarzfuchs D. Henkin Y. Shahar D. R. Witkow S. Greenberg I. (2008). Weight loss with a low-carbohydrate, Mediterranean, or low-fat diet. N. Engl. J. Med. 359, 229–241. 10.1056/NEJMoa0708681 18635428

[B184] Sharma R. P. Grayson D. R. Gavin D. P. (2008). Histone deactylase 1 expression is increased in the prefrontal cortex of schizophrenia subjects: analysis of the National Brain Databank microarray collection. Schizophr. Res. 98, 111–117. 10.1016/j.schres.2007.09.020 17961987 PMC2254186

[B185] Sicras-Mainar A. Rejas-Gutierrez J. Navarro-Artieda R. Blanca-Tamayo M. (2013). C-reactive protein as a marker of cardiovascular disease in patients with a schizophrenia spectrum disorder treated in routine medical practice. Eur. Psychiatry 28, 161–167. 10.1016/j.eurpsy.2011.07.003 21964485

[B186] Sigthorsson G. Tibble J. Hayllar J. Menzies I. Macpherson A. Moots R.. (1998). Intestinal permeability and inflammation in patients on NSAIDs. Gut 43, 506–511. 10.1136/gut.43.4.506 9824578 PMC1727292

[B187] Simopoulos A. P. (2002). The importance of the ratio of omega-6/omega-3 essential fatty acids. Biomed. Pharmacother. 56, 365–379. 10.1016/S0753-3322(02)00253-6 12442909

[B188] Singh M. M. Kay S. R. (1976). Wheat gluten as a pathogenic factor in schizophrenia. Science 191, 401–402. 10.1126/science.1246624 1246624

[B189] Solberg D. K. Bentsen H. Refsum H. Andreassen O. A. (2015). Association between serum lipids and membrane fatty acids and clinical characteristics in patients with schizophrenia. Acta Psychiatr. Scand. 132, 293–300. 10.1111/acps.12388 25597473

[B190] Soltani S. Shirani F. Chitsazi M. J. Salehi-Abargouei A. (2016). The effect of dietary approaches to stop hypertension (DASH) diet on weight and body composition in adults: a systematic review and meta-analysis of randomized controlled clinical trials. Obes. Rev. 17, 442–454. 10.1111/obr.12391 26990451

[B191] Sommer I. E. van Westrhenen R. Begemann M. J. de Witte L. D. Leucht S. Kahn R. S. (2013). Efficacy of anti-inflammatory agents to improve symptoms in patients with schizophrenia: an update. Schizophr. Bull. 40, 181–191. 10.1093/schbul/sbt139 24106335 PMC3885306

[B192] Song X. Q. Lv L. X. Li W. Q. Hao Y. H. Zhao J. P. (2009). The interaction of nuclear factor-kappa B and cytokines is associated with schizophrenia. Biol. Psychiatry 65, 481–488. 10.1016/j.biopsych.2008.10.018 19058794

[B193] Stefanovic V. Mihajlovic G. Nenadovic M. Dejanovic S. D. Borovcanin M. Trajkovic G. (2015). The effect of antipsychotic drugs on nonspecific inflammation markers in the first episode of schizophrenia. Vojnosanit. Pregl. 72, 1085–1092. 10.2298/VSP140526016S 26898032

[B194] Stokes C. Peet M. (2004). Dietary sugar and polyunsaturated fatty acid consumption as predictors of severity of schizophrenia symptoms. Nutr. Neurosci. 7, 247–249. 10.1080/10284150400010012 15682652

[B195] Strassnig M. Brar J. S. Ganguli R. (2003). Nutritional assessment of patients with schizophrenia: a preliminary study. Schizophr. Bull. 29, 393. 10.1093/oxfordjournals.schbul.a007013 14552512

[B196] Strauss G. P. Frank M. J. Waltz J. A. Kasanova Z. Herbener E. S. Gold J. M. (2011). Deficits in positive reinforcement learning and uncertainty-driven exploration are associated with distinct aspects of negative symptoms in schizophrenia. Biol. Psychiatry 69, 424–431. 10.1016/j.biopsych.2010.10.015 21168124 PMC3039035

[B197] Subramaniam M. Chong S.-A. Pek E. (2003). Diabetes mellitus and impaired glucose tolerance in patients with schizophrenia. Can. J. Psychiatry 48, 345–347. 10.1177/070674370304800512 12866342

[B198] Suddath R. L. Christison G. W. Torrey E. F. Casanova M. F. Weinberger D. R. (1990). Anatomical abnormalities in the brains of monozygotic twins discordant for schizophrenia. New Engl. J. Med. 322, 789–794. 10.1056/NEJM199003223221201 2308615

[B199] Sugawara N. Yasui-Furukori N. Sato Y. Saito M. Furukori H. Nakagami T.. (2014). Dietary patterns are associated with obesity in Japanese patients with schizophrenia. BMC Psychiatry 14:184. 10.1186/1471-244X-14-184 24947974 PMC4087244

[B200] Susce M. T. Villanueva N. Diaz F. J. de Leon J. (2005). Obesity and associated complications in patients with severe mental illnesses: a cross-sectional survey. J. Clin. Psychiatry 66, 167–173. 10.4088/JCP.v66n0203 15705001

[B201] Szkultecka-Debek M. Miernik K. Stelmachowski J. Jakovljevic M. Jukic V. Aadamsoo K.. (2016). Schizophrenia causes significant burden to patients' and caregivers' lives. Psychiatr. Danub. 28, 104–110. 27287783

[B202] Teasdale S. B. Ward P. B. Rosenbaum S. Watkins A. Curtis J. Kalucy M.. (2016). A nutrition intervention is effective in improving dietary components linked to cardiometabolic risk in youth with first-episode psychosis. Br. J. Nutr. 115, 1987–1993. 10.1017/S0007114516001033 27153205

[B203] Tedelind S. Westberg F. Kjerrulf M. Vidal A. (2007). Anti-inflammatory properties of the short-chain fatty acids acetate and propionate: a study with relevance to inflammatory bowel disease. World J. Gastroenterol. 13, 2826–2832. 17569118 10.3748/wjg.v13.i20.2826 PMC4395634

[B204] Tregellas J. R. Smucny J. Legget K. T. Stevens K. E. (2015). Effects of a ketogenic diet on auditory gating in DBA/2 mice: a proof-of-concept study. Schizophr. Res. 169, 351–354. 10.1016/j.schres.2015.09.022 26453015 PMC4827327

[B205] Tsai G. Passani L. A. Slusher B. S. Carter R. Baer L. Kleinman J. E.. (1995). Abnormal excitatory neurotransmitter metabolism in schizophrenic brains. Arch. Gen. Psychiatry 52, 829–836. 10.1001/archpsyc.1995.03950220039008 7575102

[B206] Urpi-Sarda M. Casas R. Chiva-Blanch G. Romero-Mamani E. S. Valderas-Martinez P. Salas-Salvado J.. (2012). The Mediterranean diet pattern and its main components are associated with lower plasma concentrations of tumor necrosis factor receptor 60 in patients at high risk for cardiovascular disease. J. Nutr. 142, 1019–1025. 10.3945/jn.111.148726 22535754

[B207] Usall J. Huerta-Ramos E. Labad J. Cobo J. Nunez C. Creus M.. (2016). Raloxifene as an Adjunctive Treatment for Postmenopausal Women With Schizophrenia: a 24-Week Double-Blind, Randomized, Parallel, Placebo-Controlled Trial. Schizophr. Bull. 42, 309–317. 10.1093/schbul/sbv149 26591005 PMC4753610

[B208] Vane J. R. Botting R. M. (1998). Mechanism of action of nonsteroidal anti-inflammatory drugs. Am. J. Med. 104, 2S–8S. 10.1016/S0002-9343(97)00203-9 9572314

[B209] Verkhratsky A. Steardo L. Peng L. Parpura V. (2016). Astroglia, glutamatergic transmission and psychiatric diseases. Adv. Neurobiol. 13, 307–326. 10.1007/978-3-319-45096-4_12 27885635

[B210] Vincenzi B. Stock S. Borba C. P. Cleary S. M. Oppenheim C. E. Petruzzi L. J.. (2014). A randomized placebo-controlled pilot study of pravastatin as an adjunctive therapy in schizophrenia patients: effect on inflammation, psychopathology, cognition and lipid metabolism. Schizophr. Res. 159, 395–403. 10.1016/j.schres.2014.08.021 25261882 PMC4311769

[B211] Volkow N. D. Wang G. J. Fowler J. S. Tomasi D. Baler R. (2012). Food and drug reward: overlapping circuits in human obesity and addiction. Curr. Top. Behav. Neurosci. 11, 1–24. 10.1007/7854_2011_169 22016109

[B212] von Schacky C. Fischer S. Weber P. C. (1985). Long-term effects of dietary marine omega-3 fatty acids upon plasma and cellular lipids, platelet function, and eicosanoid formation in humans. J. Clin. Invest. 76, 1626–1631. 10.1172/JCI112147 2997286 PMC424148

[B213] Vucetic Z. Reyes T. M. (2010). Central dopaminergic circuitry controlling food intake and reward: implications for the regulation of obesity. Wiley Interdiscip. Rev. Syst. Biol. Med. 2, 577–593. 10.1002/wsbm.77 20836049

[B214] Wagner A. Aizenstein H. Venkatraman V. K. Fudge J. May J. C. Mazurkewicz L.. (2007). Altered reward processing in women recovered from anorexia nervosa. Am. J. Psychiatry 164, 1842–1849. 10.1176/appi.ajp.2007.07040575 18056239

[B215] Weickert C. S. Ligons D. L. Romanczyk T. Ungaro G. Hyde T. M. Herman M. M.. (2005). Reductions in neurotrophin receptor mRNAs in the prefrontal cortex of patients with schizophrenia. Mol. Psychiatry 10, 637–650. 10.1038/sj.mp.4001678 15940304

[B216] Weickert T. W. Weinberg D. Lenroot R. Catts S. V. Wells R. Vercammen A.. (2015). Adjunctive raloxifene treatment improves attention and memory in men and women with schizophrenia. Mol. Psychiatry 20, 685–694. 10.1038/mp.2015.11 25980345 PMC4444978

[B217] Werner S. Malaspina D. Rabinowitz J. (2007). Socioeconomic status at birth is associated with risk of schizophrenia: population-based multilevel study. Schizophr. Bull. 33, 1373–1378. 10.1093/schbul/sbm032 17443013 PMC2779876

[B218] Wing R. R. Vazquez J. A. Ryan C. M. (1995). Cognitive effects of ketogenic weight-reducing diets. Int. J. Obes. Relat. Metab. Disord. 19, 811–816. 8589783

[B219] Wolever T. M. Bolognesi C. (1996). Prediction of glucose and insulin responses of normal subjects after consuming mixed meals varying in energy, protein, fat, carbohydrate and glycemic index. J. Nutr. 126, 2807–2812. 8914952 10.1093/jn/126.11.2807

[B220] Wong J. M. de Souza R. Kendall C. W. Emam A. Jenkins D. J. (2006). Colonic health: fermentation and short chain fatty acids. J. Clin. Gastroenterol. 40, 235–243. 10.1097/00004836-200603000-00015 16633129

[B221] Wong M. L. Inserra A. Lewis M. D. Mastronardi C. A. Leong L. Choo J.. (2016). Inflammasome signaling affects anxiety- and depressive-like behavior and gut microbiome composition. Mol. Psychiatry 21, 797–805. 10.1038/mp.2016.46 27090302 PMC4879188

[B222] Yang J. Chen T. Sun L. Zhao Z. Qi X. Zhou K.. (2013). Potential metabolite markers of schizophrenia. Mol. Psychiatry 18, 67–78. 10.1038/mp.2011.131 22024767 PMC3526727

[B223] Zamaria N. (2004). Alteration of polyunsaturated fatty acid status and metabolism in health and disease. Reprod. Nutr. Dev. 44, 273–282. 10.1051/rnd:2004034 15460166

[B224] Zammit S. Allebeck P. David A. S. Dalman C. Hemmingsson T. Lundberg I.. (2004). A longitudinal study of premorbid IQ score and risk of developing schizophrenia, bipolar disorder, severe depression, and other nonaffective psychoses. Arch. Gen. Psychiatry 61, 354–360. 10.1001/archpsyc.61.4.354 15066893

